# Supplementary material for: Direct observation of coordinated assembly of individual native centromeric nucleosomes
Source: EMBO J. 2023 Jul 20;42(17):e114534. doi: 10.15252/embj.2023114534 (PMC10476280; doi:10.15252/embj.2023114534)
Supplement: Supplementary file 2 — Expanded View Figures PDF [file EMBJ-42-e114534-s006.pdf]

Expanded View Figures

Figure EV1. Ndc10 residence lifetimes are longer than those of Cse4$^{CENP\text{-}A}$, which are not severely limited by photobleaching, while both are extremely stable on CEN DNA once removed from lysate.

A Example plot of Ndc10 and Cse4$^{CENP\text{-}A}$ residence pulses on CEN DNA identified via residence lifetime assays during an entire imaging sequence acquisition. Each row represents one identified CEN DNA with all identified residences shown over entire imaging sequence (2,700 s) for Ndc10 (left) and Cse4$^{CENP\text{-}A}$ (center) with merge of Ndc10 (magenta) and Cse4$^{CENP\text{-}A}$ (green) indicating ternary residences (white).

B Example images of TIRFM endpoint colocalization assays. Visualized Ndc10-mCherry on CEN DNA after 90 min incubation and removal of lysate (0 h -top panel) or after 24 h incubation at RT in imaging buffer (bottom panel) with colocalization shown in relation to identified CEN DNA in blue circles. Scale bars 3 μm. Graph shows quantification of Ndc10 endpoint colocalization on CEN DNA at 0 h and 24 h (50 ± 2.2%, 35 ± 1.7% respectively, avg ± s.d. $n = 4$ experiments, each examining ~1,000 DNA molecules from different extracts).

C Example images of TIRFM endpoint colocalization assays. Visualized Cse4$^{CENP\text{-}A}$ GFP on CEN DNA after 90 min incubation and removal of lysate (0 h—top panel) or after 24 h incubation at RT in imaging buffer (bottom panel) with colocalization shown in relation to identified CEN DNA in blue circles. Scale bars 3 μm. Graph shows quantification of Cse4$^{CENP\text{-}A}$ endpoint colocalization on CEN DNA at 0 and 24 h (45 ± 4.4%, 36 ± 1.4% respectively, avg ± s.d. $n = 4$ experiments, each examining ~1,000 DNA molecules from different extracts).

D Kaplan–Meier analysis of mCherry photobleaching events (red—median photobleaching lifetimes of 449 s ($n = 97$)) and Ndc10 residences on CEN DNA (magenta—median lifetime of 102 s ($n = 3{,}231$)). There was a significant difference between mCherry photobleaching lifetimes and Ndc10 residence lifetime survival plots (***) on CEN (two-tailed P-value of 0 as determined by log-rank test). 95% confidence intervals indicated (dashed lines), right-censored lifetimes (plus icons) were included and unweighted in survival function estimates.

E Kaplan–Meier analysis of GFP photobleaching events (yellow—median photobleaching lifetime of 447 s ($n = 86$)) and Cse4$^{CENP\text{-}A}$ residences on CEN DNA (red—median colocalization lifetime of 88 s ($n = 1{,}054$)). There was a significant difference between GFP photobleaching lifetimes and Cse4$^{CENP\text{-}A}$ residence lifetime survival plots (***) on CEN DNA (two-tailed P-value of 0 as determined by log-rank test). 95% confidence intervals indicated (dashed lines), right-censored lifetimes (plus icons) were included and unweighted in survival function estimates.

F Quantification of the proportion of short residences (< 120 s) and long residences (> 300 s) of Non-Ternary^{Ndc10} Cse4$^{CENP\text{-}A}$ residences (0.77 and 0.06 respectively, $n = 612$ over three experiments of ~1,000 DNA molecules using different extracts) or Ternary^{Ndc10} Cse4$^{CENP\text{-}A}$ (0.60 and 0.14 respectively, $n = 539$ over three experiments of ~1,000 DNA molecules using different extracts).

G Example plot of residences of Cse4$^{CENP\text{-}A}$ on CDEIII^{mut} CEN DNA per imaging sequence. Each row represents one identified CEN DNA with all identified residences shown over entire imaging sequence (2,700 s) for Cse4$^{CENP\text{-}A}$.

H Cse4$^{CENP\text{-}A}$ residence lifetimes on CDEIII^{mut} CEN DNA are reduced. Estimated survival function plots of Kaplan–Meier analysis of residence lifetimes of Cse4$^{CENP\text{-}A}$ on CEN DNA (blue—median lifetime of 82 s, $n = 1{,}419$ over three experiments of ~1,000 DNA molecules using different extracts) and residences on CDEIII^{mut} CEN DNA of Cse4$^{CENP\text{-}A}$ (red—of 52 s, $n = 471$ over three experiments of ~1,000 DNA molecules using different extracts). There was a significant difference (***) between CEN DNA and CDEIII^{mut} CEN DNA lifetime survival plots (two-tailed P-value of 0 as determined by log-rank test). 95% confidence intervals indicated (dashed lines), right-censored lifetimes (plus icons) were included and unweighted in survival function estimates.

 © 2023 The Authors

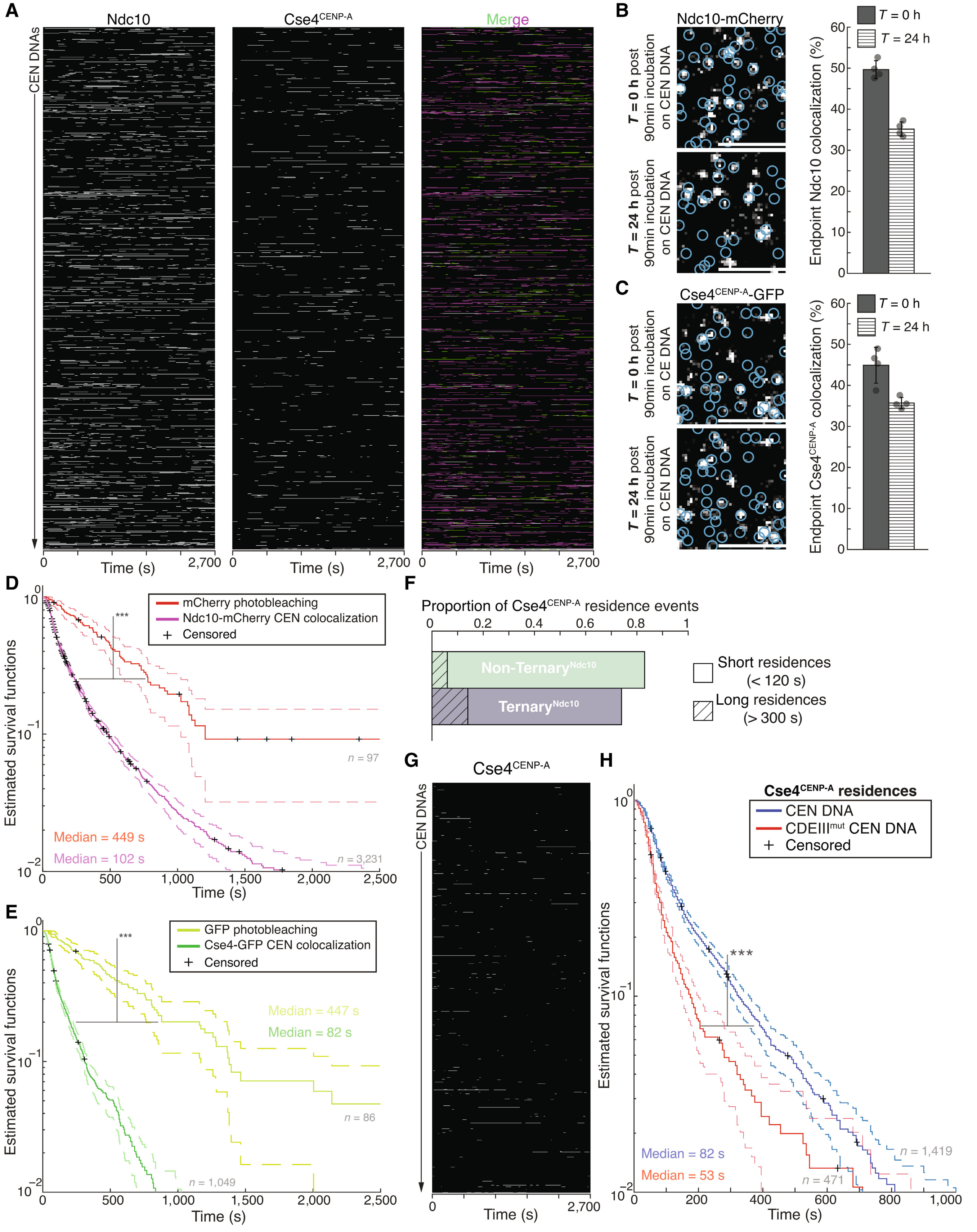
A
Ndc10
Cse4CENP-A
Merge
CEN DNAs
Time (s)
B
Ndc10-mCherry
T = 0 h post 90min incubation on CEN DNA
T = 24 h post 90min incubation on CEN DNA
T = 0 h
T = 24 h
Endpoint Ndc10 colocalization (%)
C
Cse4CENP-A-GFP
T = 0 h post 90min incubation on CE DNA
T = 24 h post 90min incubation on CEN DNA
Endpoint Cse4CENP-A colocalization (%)
D
mCherry photobleaching
Ndc10-mCherry CEN colocalization
+ Censored
Median = 449 s
Median = 102 s
n = 97
n = 3,231
Estimated survival functions
Time (s)
E
GFP photobleaching
Cse4-GFP CEN colocalization
+ Censored
Median = 447 s
Median = 82 s
n = 86
n = 1,049
F
Proportion of Cse4CENP-A residence events
Non-TernaryNdc10
TernaryNdc10
Short residences (< 120 s)
Long residences (> 300 s)
G
Cse4CENP-A
H
Cse4CENP-A residences
CEN DNA
CDEIIImut CEN DNA
+ Censored
Median = 82 s
Median = 53 s
n = 1,419
n = 471

Figure EV1.

Figure EV2. Scm3^{HJURP} has shorter residence lifetimes than Cse4$^{CENP\text{-}A}$ on CEN DNA.

A Example plot of Cse4$^{CENP\text{-}A}$ and Scm3^{HJURP} residences on CEN DNA per imaging sequence. Each row represents one identified CEN DNA with all identified residences shown over entire imaging sequence (2,700 s) Cse4$^{CENP\text{-}A}$ (left) and Scm3^{HJURP} (center) with merge indicating Cse4$^{CENP\text{-}A}$ (green), Scm3^{HJURP} (magenta) and ternary residences (white).

B Estimated survival function plots of Kaplan–Meier analysis of Cse4$^{CENP\text{-}A}$ residence lifetimes on CEN DNA (green—median lifetime of 82 s, n = 1,419 over three experiments of ~1,000 DNA molecules using different extracts), and residence lifetimes of Scm3^{HJURP} on CEN DNA (magenta—median lifetime of 61 s, n = 1,115 over three experiments of ~1,000 DNA molecules using different extracts). 95% confidence intervals indicated (dashed lines). Significant difference (***) between Cse4$^{CENP\text{-}A}$ and Scm3^{HJURP} residence survival plots (two-tailed P-value of 9.1e-14 as determined by log-rank test), right-censored lifetimes (plus icons) were included and unweighted in survival function estimates.

C Quantification of the estimated off-rates of Scm3^{HJURP} that never formed a ternary residence (Non-Ternary^{Cse4}) and of Scm3^{HJURP} after ternary residence with Cse4$^{CENP\text{-}A}$ (Ternary^{Cse4}) on CEN DNA (114 s ± 13 s and 111 s ± 19 s respectively, avg ± s.d. n = 2,050 over three experiments of ~1,000 DNA molecules using different extracts). No significant difference between off-rates (n.s.) with a P-value of 0.87 as determined by two-tailed unpaired t-test.

D Estimated survival function plots of Kaplan–Meier analysis of the lifetimes of Ternary^{Cse4} Scm3^{HJURP} residences on CEN DNA (purple—median lifetime of 69 s, n = 311 over three experiments of ~1,000 DNA molecules using different extracts) and Non-Ternary^{Cse4} Scm3^{HJURP} residences on CEN DNA (green—of 63 s, n = 1,279 over three experiments of ~1,000 DNA molecules using different extracts). No significant difference (n.s.) between Ternary^{Cse4} and Non-Ternary^{Cse4} survival plots (two-tailed P-value of 0.75 as determined by log-rank test). 95% confidence intervals indicated (dashed lines), right-censored lifetimes (plus icons) were included and unweighted in survival function estimates.

E Timing of all observed Cse4$^{CENP\text{-}A}$ and Scm3^{HJURP} ternary residence events. The proportion when Scm3^{HJURP} precedes Cse4$^{CENP\text{-}A}$ (Scm3^{HJURP} First) is 0.46, followed by 0.34 when Cse4$^{CENP\text{-}A}$ precedes Scm3^{HJURP} (Scm3^{HJURP} Last), with a proportion of 0.20 co-arrival events (Co-arrival—defined as residence initiation within 5 s of each, n = 305 over three experiments of ~1,000 DNA molecules using different extracts).

F Estimated survival function plots of Kaplan–Meier analysis of the lifetimes of Scm3^{HJURP}-First-Ternary^{Scm3} Cse4$^{CENP\text{-}A}$ residences on CEN DNA (purple—median lifetime of 108 s, n = 142 over three experiments of ~1,000 DNA molecules using different extracts) and Scm3^{HJURP}-Last-Ternary^{Scm3} Cse4$^{CENP\text{-}A}$ residences on CEN DNA (green—of 133 s, n = 102 over three experiments of ~1,000 DNA molecules using different extracts). No significant difference (n.s.) between Scm3^{HJURP}-First and Scm3^{HJURP}-Last survival plots (two-tailed P-value of 0.59 as determined by log-rank test). 95% confidence intervals indicated (dashed lines), right-censored lifetimes (plus icons) were included and unweighted in survival function estimates.

© 2023 The Authors

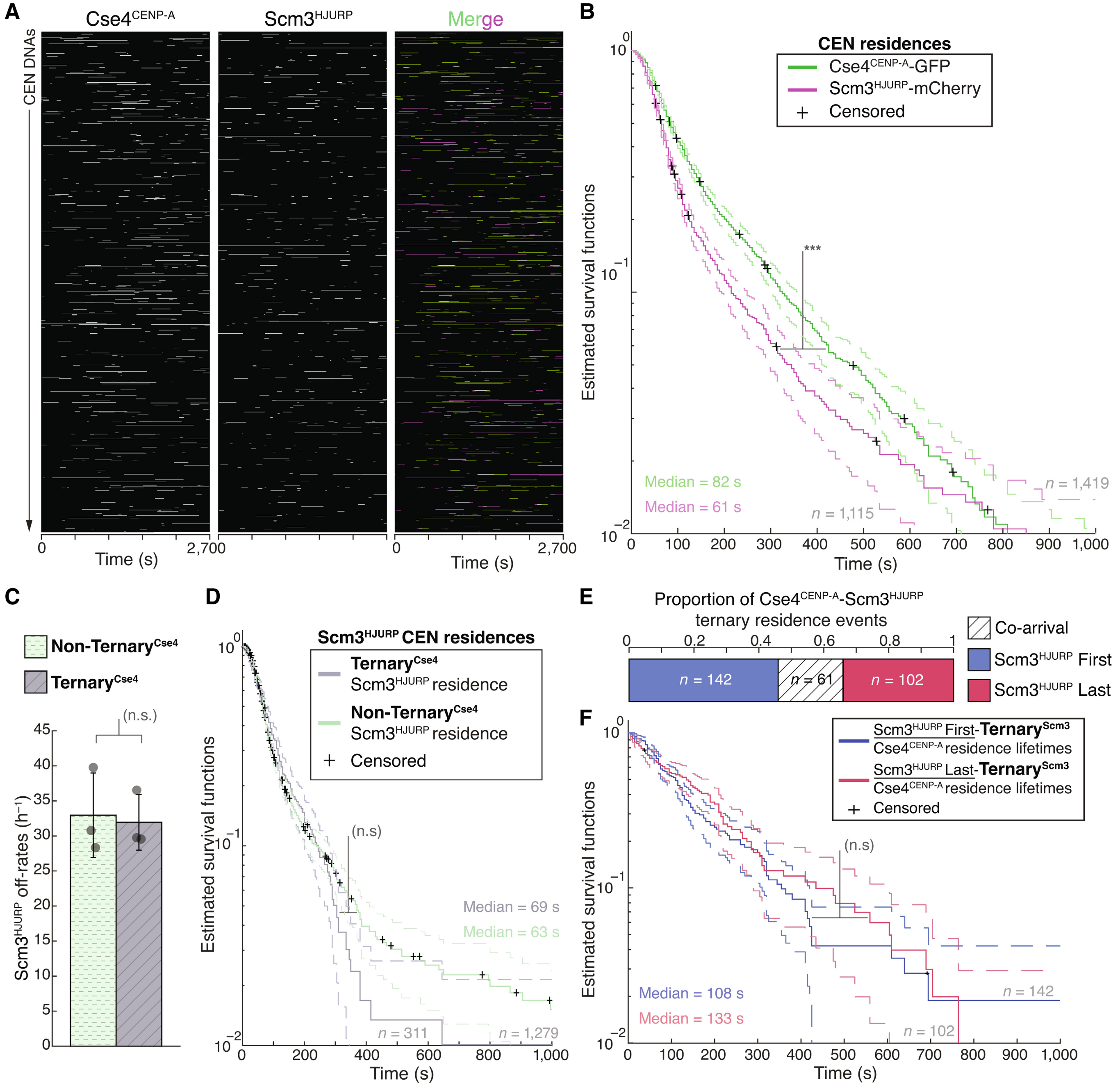
A
Cse4CENP-A
Scm3HJURP
Merge
CEN DNAs
0
2,700
Time (s)
B
CEN residences
Cse4CENP-A-GFP
Scm3HJURP-mCherry
Censored
Estimated survival functions
***
Median = 82 s
Median = 61 s
n = 1,115
n = 1,419
Time (s)
C
Non-TernaryCse4
TernaryCse4
(n.s.)
Scm3HJURP off-rates (h−1)
D
Scm3HJURP CEN residences
TernaryCse4 Scm3HJURP residence
Non-TernaryCse4 Scm3HJURP residence
Censored
Estimated survival functions
(n.s)
Median = 69 s
Median = 63 s
n = 311
n = 1,279
Time (s)
E
Proportion of Cse4CENP-A-Scm3HJURP ternary residence events
Co-arrival
Scm3HJURP First
Scm3HJURP Last
n = 142
n = 61
n = 102
F
Scm3HJURP First-TernaryScm3 Cse4CENP-A residence lifetimes
Scm3HJURP Last-TernaryScm3 Cse4CENP-A residence lifetimes
Censored
Estimated survival functions
(n.s)
Median = 108 s
Median = 133 s
n = 142
n = 102
Time (s)

Figure EV2.

Figure EV3. Cse4$^{CENP\text{-}A}$ interacts transiently with CDEIII^{mut} CEN DNA with or without its chaperone Scm3^{HJURP}.

A Example plot of residences of Cse4$^{CENP\text{-}A}$ and Scm3^{HJURP} on CDEIII^{mut} CEN DNA per imaging sequence. Each row represents one identified CEN DNA with all identified residences shown over entire imaging sequence (2,700 s) for Cse4$^{CENP\text{-}A}$ (left) and Scm3^{HJURP} (center) with merge indicating Cse4$^{CENP\text{-}A}$ (green), Scm3^{HJURP} (magenta) and ternary residences (white).

B Scm3^{HJURP} residences are longer on CDEIII^{mut} CEN DNA. Estimated survival function plots of Kaplan–Meier analysis of Scm3^{HJURP} residence lifetimes on CEN DNA (blue—median lifetime of 61 s, n = 1,115 over three experiments of ~1,000 DNA molecules using different extracts), and residence lifetimes of Scm3^{HJURP} on CDEIII^{mut} CEN DNA (red—median lifetime of 75 s, n = 2,269 over three experiments of ~1,000 DNA molecules using different extracts). There was a significant difference (***) between CEN DNA and CDEIII^{mut} CEN DNA lifetime survival plots (two-tailed P-value of 5.13e-13 as determined by log-rank test). 95% confidence intervals indicated (dashed lines), right-censored lifetimes (plus icons) were included and unweighted in survival function estimates.

C Cse4$^{CENP\text{-}A}$ residence lifetimes are similar without Scm3^{HJURP} a on CDEIII^{mut} CEN DNA. Estimated survival function plots of Kaplan–Meier analysis of ternary residence lifetimes of Ternary^{Scm3} Cse4$^{CENP\text{-}A}$ residences on CDEIII^{mut} CEN DNA (blue—median lifetime of 45 s, n = 66 over three experiments of ~1,000 DNA molecules using different extracts) and Non-Ternary^{Scm3} Cse4$^{CENP\text{-}A}$ residences on CDEIII^{mut} CEN DNA (red—of 52 s, n = 433 over three experiments of ~1,000 DNA molecules using different extracts). There was no significant difference (n.s.) between Non-Ternary^{Scm3} and Ternary^{Scm3} survival plots (two-tailed P-value of 0.27 as determined by log-rank test). 95% confidence intervals indicated (dashed lines), right-censored lifetimes (plus icons) were included and unweighted in survival function estimates.

© 2023 The Authors

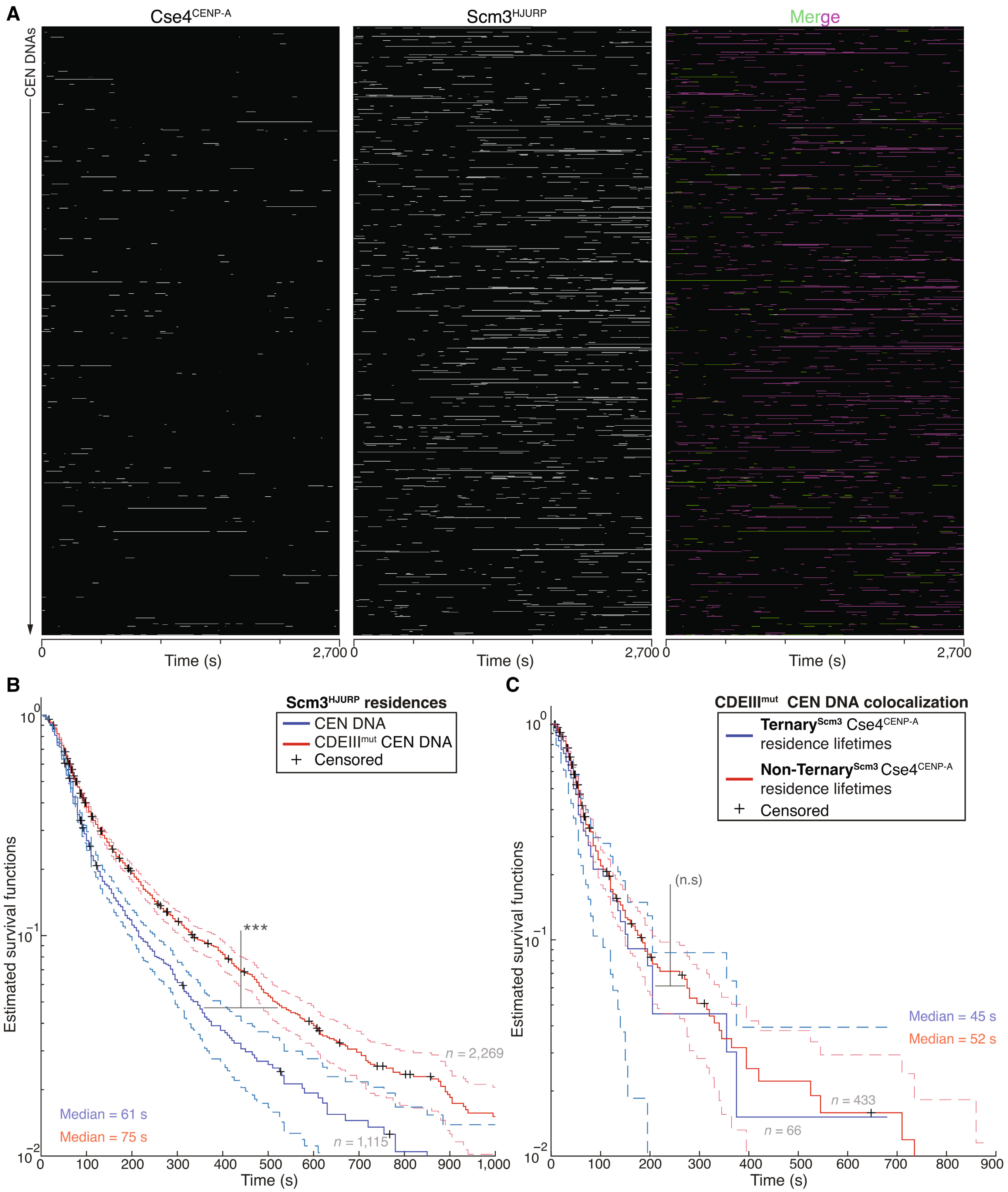
A
Cse4CENP-A
Scm3HJURP
Merge
CEN DNAs
Time (s)
0
2,700
B
Scm3HJURP residences
CEN DNA
CDEIIImut CEN DNA
Censored
Estimated survival functions
***
n = 2,269
n = 1,115
Median = 61 s
Median = 75 s
Time (s)
C
CDEIIImut CEN DNA colocalization
TernaryScm3 Cse4CENP-A residence lifetimes
Non-TernaryScm3 Cse4CENP-A residence lifetimes
Censored
Estimated survival functions
(n.s)
Median = 45 s
Median = 52 s
n = 433
n = 66
Time (s)

Figure EV3.

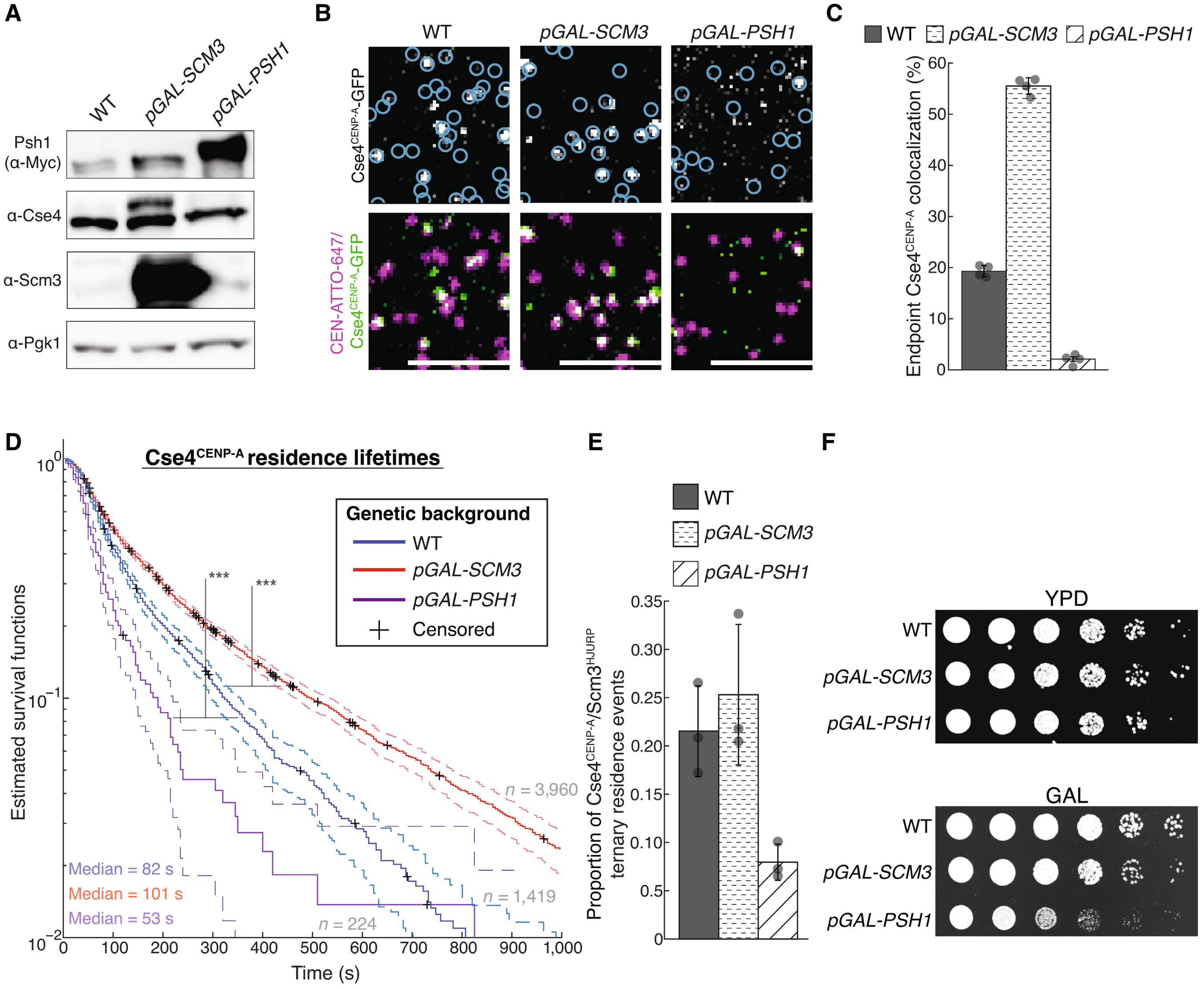

Figure EV4. Scm3^{HJURP}-Cse4^{CENP-A} complex is limiting for stable centromeric association of Cse4^{CENP-A}.

A Immunoblot analysis of whole cell extracts from WT, *pGAL-SCM3* and *pGAL-PSH1* cells using indicated antibodies (all panels cropped from the same blot).

B Example images of TIRFM endpoint colocalization assays. Top panels show visualized Cse4^{CENP-A} -GFP on CEN DNA in extracts from a WT genetic background (top-left panel) or extracts containing overexpressed Scm3^{HJURP} (*pGAL-SCM3*, top-middle panel) or overexpressed Psh1 (*pGAL-PSH1*, top-right panel) with colocalization shown in relation to identified CEN DNAs in blue circles. Bottom panels show overlay of DNA channel (magenta) with Cse4^{CENP-A} -GFP (green). Scale bars 3 μm.

C Quantification of endpoint colocalization of Cse4^{CENP-A} on CEN DNA in extracts from a WT genetic background, extracts that contain overexpressed Scm3^{HJURP} or extracts that contain overexpressed Psh1 (19 ± 1.1%, 56 ± 1.6% and 2.1 ± 0.4% respectively, avg ± s.d. n = 4 experiments, each examining ~1,000 DNA molecules from different extracts).

D Estimated survival function plots of Kaplan–Meier analysis of residence lifetimes of Cse4^{CENP-A} on CEN DNA in extracts from WT genetic background (blue—median lifetime of 82 s, n = 1,419 over three experiments of ~1,000 DNA molecules using different extracts), or from extracts that contain overexpressed Scm3^{HJURP} (red—median lifetime of 101 s, n = 3,960 over three experiments of ~1,000 DNA molecules using different extracts) or extracts that contain overexpressed Psh1 (purple—median lifetime of 52 s, n = 224 over three experiments of ~1,000 DNA molecules using different extracts). Significant difference (***) between survival plots in WT extracts compared to those overexpressing Psh1 (two-tailed P-value of 8.9e-11 as determined by log-rank test) or Scm3^{HJURP} (two-tailed P-value of 0 as determined by log-rank test). 95% confidence intervals indicated (dashed lines), right-censored lifetimes (plus icons) were included and unweighted in survival function estimates.

E Proportion of ternary residences of Cse4^{CENP-A} with Scm3^{HJURP} on CEN DNA for WT extracts (0.22 ± 0.05, avg ± s.d. n = 1,419 over three experiments of ~1,000 DNA molecules using different extracts), extracts containing overexpressed Scm3^{HJURP} (0.25 ± 0.07, avg ± s.d. n = 3,960 over three experiments of ~1,000 DNA molecules using different extracts) and extracts containing overexpressed Psh1 (0.08 ± 0.02, avg ± s.d. n = 224 over three experiments of ~1,000 DNA molecules using different extracts).

F Serial five-fold dilutions of the following yeast strains were plated and grown 2 days on YPD and 3 days on galactose (GAL) at 23° C: WT (SBY21441), *pGAL-SCM3* (SBY21443), and *pGAL-PSH1* (SBY20836).

 © 2023 The Authors

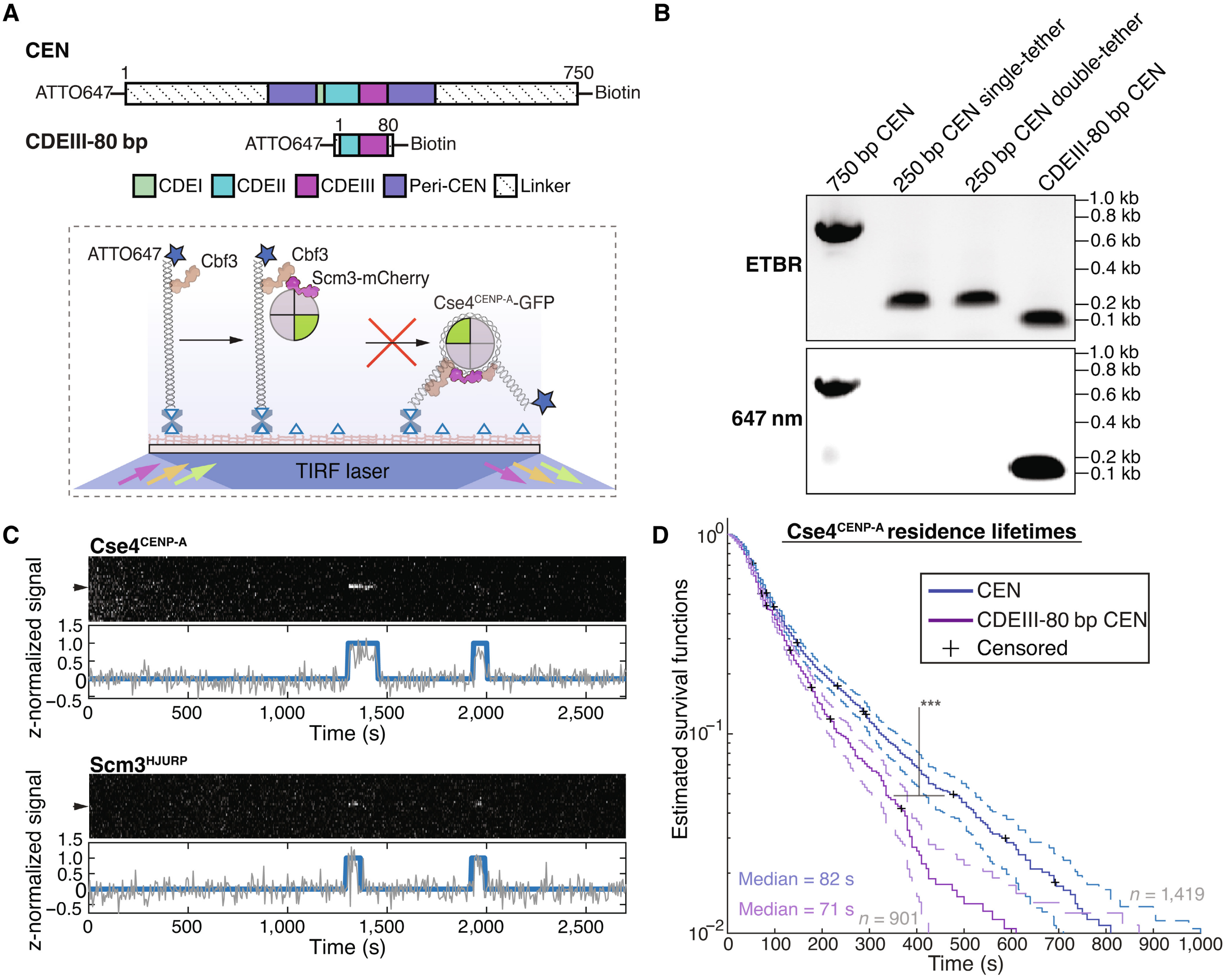

Figure EV5. Cse4^{CENP-A} residence lifetimes are significantly reduced on CDEIII-80 bp mutant CEN DNA.

A Schematic of overview of CDEIII-80 bp mutant CEN DNA, the canonical CEN DNA is shortened to 80 bp to prevent nucleosome formation and then similarly functionalized to the coverslip via a single biotin at the 5′ end and functionalized with an organic dye at the free 3′ end.

B CEN assembly templates including WT 750 bp CEN DNA, 250 bp single-tether CEN DNA, 250 bp double-tethered CEN DNA and CDEIII-80 bp CEN DNA as visualized via EtBr (top panel) or 647 nm excitation (bottom panel) on a 1% agarose gel.

C Representative colocalization traces of Cse4^{CENP-A} and Scm3^{HJURP} on a single CDEIII-80 bp CEN DNA. Top panel includes kymograph of Cse4^{CENP-A} (top-488 nm) in relation to single identified CEN DNA (arrow), with normalized intensity trace (gray-bottom) as well as identified colocalization pulses (blue). Bottom panel includes kymograph of Scm3^{HJURP} (bottom-568 nm) in relation to the same identified CEN DNA (arrow), with normalized intensity trace (gray-bottom) as well as identified colocalization pulse (blue). Cases where identified pulses in Scm3^{HJURP} and Cse4^{CENP-A} coincide represent observed colocalization of both proteins on single CDEIII-80 bp CEN DNA. Images acquired every 5 s with normalized fluorescence intensity shown in arbitrary units.

D Estimated survival function plots of Kaplan–Meier analysis of all identified CEN DNA colocalization events of Cse4^{CENP-A} (blue—median lifetime of 82 s, n = 1,619 over three experiments of ~1,000 DNA molecules using different extracts) and identified colocalization events on CDEIII-80 bp CEN DNA of Cse4^{CENP-A} (red—median lifetime of 71 s, n = 901 over three experiments of ~1,000 DNA molecules using different extracts). Significant difference (***) between CEN DNA and CDEIII-80 bp CEN DNA lifetime survival plots (two-tailed P-value of 1.0e-6 as determined by log-rank test). 95% confidence intervals indicated (dashed lines), right-censored lifetimes (plus icons) were included and unweighted in survival function estimates.

© 2023 The Authors
