# Supplementary material for: Direct observation of coordinated assembly of individual native centromeric nucleosomes
Source: EMBO J. 2023 Jul 20;42(17):e114534. doi: 10.15252/embj.2023114534 (PMC10476280; doi:10.15252/embj.2023114534)
Supplement: Supplementary file 4 — Movie EV2 [file EMBJ-42-e114534-s002.zip › Movie EV2.rtf]

Movie EV2. Ndc10 association with CEN3 DNA after stable Cse4CENP-A recruitment. Movie showing the colocalization to single CEN3 DNA (647nm, center of left panel) of Cse4CENP‑-GFP (488 nm, midle panel) and Ndc10-mCherry (568 nm, right panel). This movie corresponds to Figure 2C - Ndc10 Last. Scale bar 3 m.
