# Supplementary material for: Direct observation of coordinated assembly of individual native centromeric nucleosomes
Source: EMBO J. 2023 Jul 20;42(17):e114534. doi: 10.15252/embj.2023114534 (PMC10476280; doi:10.15252/embj.2023114534)
Supplement: Supplementary file 5 — Movie EV3 [file EMBJ-42-e114534-s008.zip › Movie EV3.rtf]

Movie EV3: Stable Cse4CENP-A recruitment coincides with ternary CEN3 DNA residence with Scm3HJURP. Movie showing the colocalization to single CEN3 DNA (647nm, left panel) of Cse4CENPA-GFP (488 nm, middle panel) and Scm3HJURP-mCherry (568 nm, right panel). This movie corresponds to Figure 3A. Scale bar 3 m.
