# Supplementary material for: Direct observation of coordinated assembly of individual native centromeric nucleosomes
Source: EMBO J. 2023 Jul 20;42(17):e114534. doi: 10.15252/embj.2023114534 (PMC10476280; doi:10.15252/embj.2023114534)
Supplement: Supplementary file 6 — Movie EV4 [file EMBJ-42-e114534-s001.zip › Movie EV4.rtf]

Movie EV4: Double-tethered 250 bp CEN3 DNA fails to stably recruit Cse4CENPA. Movie showing the colocalization to single Ndc10-mCherry on single tethered CEN3 DNA (568 nm, top left panel) of Cse4CENPA-GFP (488 nm, top right panel) or on double tethered CEN3 DNA (568 nm, center of bottom left panel) of Cse4CENPA-GFP (488 nm, bottom right panel). This movie corresponds to Figure 4E (top and middle panels). Scale bar 3 m.
