# Supplementary material for: Direct observation of coordinated assembly of individual native centromeric nucleosomes
Source: EMBO J. 2023 Jul 20;42(17):e114534. doi: 10.15252/embj.2023114534 (PMC10476280; doi:10.15252/embj.2023114534)
Supplement: Supplementary file 7 — Movie EV5 [file EMBJ-42-e114534-s003.zip › Movie EV5.rtf]

Movie EV5: 80 bp CDEIII mutant CEN DNA fails to stably recruit Cse4CENPA. Movie showing the colocalization to single 80 bp CDEIII mutant CEN DNA (647nm, center of left panel) of Cse4CENP-A-GFP (488 nm, right panel). This movie corresponds to Figure 4E (bottom panel). Scale bar 3 m.
