# Supplementary material for: Direct observation of coordinated assembly of individual native centromeric nucleosomes
Source: EMBO J. 2023 Jul 20;42(17):e114534. doi: 10.15252/embj.2023114534 (PMC10476280; doi:10.15252/embj.2023114534)
Supplement: Supplementary file 9 [file EMBJ-42-e114534-s009.pdf]

THE EMBO JOURNAL

Direct observation of coordinated assembly of individual native centromeric nucleosomes

Andrew Popchock, Joshua Larson, Julien Dubrulle, Charles Asbury, and Sue Biggins
DOI: 10.15252/embj.2023114534

Corresponding author(s): Sue Biggins (sbiggins@fhcrc.org)

Review Timeline:

Transfer from Review Commons:	16th May 23
Editorial Decision:	23rd Jun 23
Revision Received:	27th Jun 23
Accepted:	4th Jul 23

Review COMMONS

Editor: Hartmut Vodermaier

Transaction Report: This manuscript was transferred to The EMBO JOURNAL following peer review at Review Commons.

Review #1

1. Evidence, reproducibility and clarity:

Evidence, reproducibility and clarity (Required)

Summary

This work presents a novel experimental setup to explore native centromeric nucleosome formation at a single molecule level with a high degree of temporal resolution. It combines TIRF microscopy with an immobilized fluorescent yeast CEN3 DNA sequence. Binding of centromere proteins was probed by using whole-cell lysates produced from various mutant yeast strains expressing fluorescently tagged centromere proteins.
This system was used to investigate the recruitment of Cse4 (yeast CENP-A), the histone H3 variant which defines centromeres, to the native yeast CDE3 sequence. Consistent with previous studies, the authors identified a requirement for Ndc10, which binds the CDE3 sequence with high efficiency, in Cse4 recruitment. Colocalization of Ndc10 with Cse4 significantly increases the stability of the Cse4-CDE3 interaction, and Ndc10 binding predominantly precedes Cse4 recruitment. Secondly, they investigated the impact of Scm3 (yeast equivalent of HJURP) on Cse4 stability and found that Scm3 is not required for transient short-term associations between the DNA and Cse4 but is needed for longer more stable interactions between Cse4 & the CDE3 sequence. Overexpression of Scm3 & its negative regulator Psh1 also showed that Scm3 availability is the rate-limiting factor in stable Cse4 associations with DNA. However, these interactions were abolished when the CDE3 DNA sequence was topologically restricted by tethering both ends to the streptavidin-coated coverslip suggesting that the formation of a nucleosome is essential for stable binding of Cse4 to CEN3. The sequence-specific DNA binding kinetochore proteins ChI4 & Okp1 were also found to improve the lifetime of Cse4-CDE3 interactions suggesting a stabilizing interaction. Finally, the impact of A/T runs on nucleosome formation was examined using a library of scrambled CDEII sequences, with approximately native levels As & Ts but shorter runs of the 2 nucleotides. Previous studies found a correlation between the length of AT runs and the incidence of chromosome missegregation. They now extend this correlation to a decreased ability of DNA to stably recruit Cse4. Finally, experiments with other sequences including the Wisdom601 sequence suggest that A/T runs along with CBF3 binding inhibit the recruitment of histone H3 to the CDEII sequence which is overcome by the interactions between Cse4 and Scm3.

Assessment

This is an insightful, well-executed study of a high technical standard. While several observations confirm or further validate previous findings in vivo (such as the requirement for Ndc10 and Scm3 for Cse4 assembly), this work adds a better understanding of the dynamics of Cse4 requirement and the role of Scm3 in assembly and the role of CCAN components in stabilizing Cse4. Figures are well laid out and methods are clearly described. In general, the claims are supported by the data.
The development of their single-molecule setup to assay Cse4 nucleosome assembly is a promising tool for future work and for this reason work reporting. In short, I believe this paper contains exciting developments in the understanding of the specific mechanism and temporal dynamics of the formation of a Cse4 nucleosome on its native DNA and the interactions which underpin its stability in vitro and in vivo.

Major comments

I have several comments and suggestions for further analysis of the data. I also have suggestions for additional experiments, but I would stress that none of these are essential for publication.

Figure 2E. This shows the residence time of Cse4 without Ndc10 association. How does this compare to the residence time on mutant CEN3 (Supplemental Figure 1). It looks like Cse4 still binds to CEN3 with some specificity even in the absence of Ndc10. Does this suggest that Cse4 has some intrinsic ability to recognize CEN3? Alternatively, Ndc10 is still required for Cse4 binding but is below detection in the Cse4-alone residence lifetimes. Ideally, the authors would compare this with Cse4 binding in an Ndc10 mutant.

Figure 3 explores the very interesting relationship between Scm3 dynamics and Cse4 binding but I feel that the data is not fully flushed out.
A key finding is that Cse4 can potentially bind to CEN DNA prior to engaging with Scm3 to be incorporated. This predicts that Cse4 should bind first and then colocalize with Scm3. Can this be detected in the timing of the traces? How often does Scm3 bind to already prebound Cse4 and does this correlate with Cse4 residing longer?

A related and perhaps even more interesting point is whether Scm3 is involved in "loading" of Cse4. If so, then one would expect that once Cse4 is assembled into nucleosomes it should be stable, even if Scm3 leaves. Can the authors extract this from the data? Alternatively, it is possible that Scm3 remains associated to Cse4 to maintain the nucleosome which would imply a more extended role for Scm3 apart from assembly alone. It would be interesting if information on this can be extracted

from the data.

Even in the presence of Scm3 and CCAN components, Cse4 appears to have a limited lifetime in the in vitro assay compared to in vivo stability. The authors should speculate on whether activities exist in their extract that actively disassembles nucleosomes. Perhaps ATP could be depleted to inactivate remodellers?

For Figure 6, it is not clear why AT-track mutants of CDEII are labeled as genetically stable and genetically unstable. This is confusing as the "genetically stable" show a more than 10-fold increase in chromosome loss rates. Perhaps these can be renamed into "unstable" and "very unstable" or "weak" and "strong" mutants, just to make clear that these classes are both poorer than wild type.

Finally, it would be wonderful to include data to assess whether a full Cse4 nucleosome is assembled or a partial nucleosome e.g. just Cse4/H4 tetrasomes. This could be done by tracking the accumulation of H2A or H2B at the CEN3. This would give further insight into what step Scm3 catalyses.

Minor comments:

Typo on page 5, line 1 "nucleosom" missing an e.

Kaplan-Meyer should be spelled Kaplan-Meier

The term "censored" is mentioned across many figures but comes up just ones in the methods where it is not clearly explained. Perhaps this could be clarified in the legend.

The abstract states that Cse4 can arrive at the centromere without its chaperone. More likely, Cse4 is in complex with other chaperones that may allow it to bind. Perhaps the abstract can be modified to read "Cse4 can arrive at the centromere without its dedicated centromere-specific chaperone Scm3..."

Related to this point, the discussion states the possibility that Cse4 can initially bind to CEN3 via other more general chaperones. However, it should be acknowledged that transient Cse4 binding in their assay may simply occur through mass action due to high concentrations of CEN3 DNA. In vivo, this transient binding may not be that relevant.

2. Significance:

Significance (Required)

This paper offers new insight into the assembly of yeast centromeres with a focus on the role of the Chaperone Scm3 in the assembly of the centromere-specific histone Cse4. This is still a poorly understood process and the authors offer an elegant in vitro system to study this and have presented new insights. For this reason, this is study is of interest to a broad readership in the area of mitosis and chromosome structure. The advance is strong at the technical level but also new insight is provided particularly in the role of Scm3 and the nature of centromeric DNA in centromeric chromatin assembly. Overall a strong, high-quality paper.

3. How much time do you estimate the authors will need to complete the suggested revisions:

Estimated time to Complete Revisions (Required)

(Decision Recommendation)

Less than 1 month

4. *Review Commons* values the work of reviewers and encourages them to get credit for their work. Select 'Yes' below to register your reviewing activity at Web of Science Reviewer Recognition Service (formerly Publons); note that the content of your review will not be visible on Web of Science.

Web of Science Reviewer Recognition

Yes

Review #2

1. Evidence, reproducibility and clarity:

Evidence, reproducibility and clarity (Required)

Centromeres drive chromosome inheritance from one cell generation to the next, so understanding their nature is of utmost importance in biology. The assembly of centromeric chromatin is of outstanding interest since defects in this step impair faithful genetic inheritance. Popchock et al investigate the molecular mechanisms of Cse4(CENPA) deposition and stabilization on a native budding yeast centromeric DNA. Using TIRMF (Total Internal Reflection Fluorescent Microscopy) enabled single molecule visualization of de novo Cse4(CENPA) nucleosome formation from a yeast cellular extract. The centromeric DNA used in this study was derived from PCR amplification of plasmids containing the native yeast point centromere from chromosome 3 (CEN3 DNA) sequences containing the CDEI, CDEII, CDEII, flanking pericentromeric DNA and linker DNA totaling ~750bp. In the PCR preparation, the CEN3 DNA contains a 5'-fluorescent dye and a biotinylated 3'-end, and was tethered to a functionalized (streptavidin) slide for TIRFM. The yeast extract was extracted from cells arrested in mitosis. This system is a novel application of a single molecule to study Cse4(CENPA) formation on centromeric DNA.

Using this system, the authors observed coordinated Cse4(CENPA) deposition on the CEN3 DNA, reporting inherent, transient colocalization of Cse4(CENPA) with CEN3 DNA. Stable Cse4(CENPA) colocalization on the CEN3 DNA is correlated with the Cse4(CENPA) chaperone Smc3(HJURP), and an ability for nucleosome formation on the CEN3 DNA. Further stabilization of Cse4(CENPA) was shown to depend on the DNA binding CCAN protein chl4(CENPN) and okp1(CENPQ) which dimerizes with the DNA/CENPA binding Ame1(CENPU).

Using this single molecule system they also demonstrated a role for the A/T run (>4) content in the CDEII as specifically important for Cse4(CENPA) deposition on CEN3 DNA. Cse4(CENPA) colocalization was preferred on the native CDEII sequence, relative to mutant CDEII sequences with similar A/T content but variable homopolymeric runs.

Major comments:

1. Figure S1A-D seem like some of the most compelling data in the paper to bolster the rigor of their experimental setup. There appears to be plenty of space to include these data in the main figure set in Figure 1 after panel D. The authors would be well served to consider moving S1A-D somewhere in the main figure set.
2. The authors conclude that Ndc10 recruits HJURP(Smc3) to the yeast point centromeres. If this is the case, can the TIRFM assay measure ternary residence lifetimes complexes between Ndc10/HJURP(Smc3)/CenDNA?
3. Throughout the manuscript short- and long- term residence lifespans are mentioned, referencing the figures containing lines with lengths depicting residence times. This is

a qualitative reference to short and long residences. Can the authors provide a graph for short-term (<120 s) and long-term (> 300 s) residence life-spans for, CENPA alone, CENPA/Ndc10, and CENPA/HJURP on CEN3 DNA? Or some figure similar to Figure 3C, but reporting the proportion of short-term vs long-term residence?
4. The choice of CCAN components for analysis in Fig. 5 is interesting, but many readers may be curious why Mif2 wasn't selected for disruption, since it has such a cozy placement with CENP-A and CEN DNA. Can choice to not include Mif2 mutants/degrons be mentioned/justified in the text (unless, even better yet, they choose to address Mif2 role directly in new experimentation)?

Minor comments:

5. Are these whole cell extracts (WCE) DNA-free? I'm curious if there is any competition from endogenous DNA from the yeast cellular extract.
6. In relation to Mif2 and comment #4 above, do the authors make any connection to their results with synthetic nucleosome sequences not being conducive to yeast centromere formation with the prior observation (Allu et al 2019) using recombinant components that the human version of Mif2 more easily saturates its binding sites on CENP-A nucleosomes when they are assembled with natural centromere DNA rather than the Widom 601 sequence?
7. Providing a gel (or other measure) of the DNA templates (750, 250, and 80 bp) used in TIRFM assay would be nice to show to confirm the designed size of the pre-tethered DNA.
8. Some of the references to figures/figure panels in the main text do not match the figures. (discussion, pg 16, paragraph 1 & 2;pg 18, paragraph 1).

2. Significance:

Significance (Required)

This work demonstrates the dynamics of Cse4(CENPA) coordination with Smc3(HJURP) to form nucleosomes on point centromeric DNA, and the necessity for homopolymeric A/T runs. It is a truly impressive piece of work that makes sense of findings from prior genetics experiments. Then it extends the understanding and clarifies the role of both centromere proteins and DNA sequence. The quantitative and powerful single molecule-based experimentation, the high importance of the subject matter, and its connection to studies using yeast genetics, will make this work, upon modest improvements (see section A of this review), of outstanding interest to an extremely broad audience of biologist.

My relevant expertise keywords: centromeres, nucleosomes, biochemical reconstitution, chromosome engineering

3. How much time do you estimate the authors will need to complete the suggested revisions:

Estimated time to Complete Revisions (Required)

(Decision Recommendation)

Between 1 and 3 months

4. *Review Commons* values the work of reviewers and encourages them to get credit for their work. Select 'Yes' below to register your reviewing activity at Web of Science Reviewer Recognition Service (formerly Publons); note that the content of your review will not be visible on Web of Science.

Web of Science Reviewer Recognition

No

Review #3

1. Evidence, reproducibility and clarity:

Evidence, reproducibility and clarity (Required)

The manuscript "Single molecule visualization of native centromeric nucleosome formation reveals coordinated deposition by kinetochore proteins and centromere DNA sequence" by Popchock and colleagues describes a new high-throughput single-molecule technique that combines both in vitro and in vivo sample sources. Budding yeast centromeres are genetically defined centromeres, which makes them ideal for studying short DNA segments at the single-molecule level. By flowing in whole cell lysates, Cse4 nucleosomes can form under near physiological conditions. Two

analytical experiments were performed: endpoint and time-lapse. In the former case, nucleosomes were allowed to form within 90 minutes and the latter case, nucleosome formation was tracked for up to 45 minutes. In addition, well described genetic mutants were used to assess the stability of Cse4 nucleosomes, as well as different DNA sequences (this we particularly liked- well done). Overall, this is a very interesting technique with potential to be useful for studying any DNA-based effect, ranging from DNA repair to kinetochore assembly. This is strong and impactful work, and the potential this kind of microscopy has for solving kinetic problems in the field. We think it's worthy of publication after revising technical and experimental concerns that would elevate the ms significantly.

Major comments:

Statistics are highly recommended for all the data in the ms.

- At what rate is data collected in the TIRFM setup. For clarity for the reader, it is important to provide imaging details for time-lapse. What is the impact of photobleaching on the stability of the fluorophore signal? Please clarify.
- The power of single-molecule technique is precisely that such data can be made quantitative. Indeed, the Kaplan-Meyer graphs do show nice quantitative results. Unfortunately, in the text few quantitative measurements are reported. In fact, the Kaplan-Meyer graphs can be interpreted in a quantitative manner such as probability of residency time. Although in most cases the statistical significance between two conditions can be expected, this is not formally calculated and shown. What is the 50% survival time of Cse4 alone or with Ndc10, for instance? This manuscript would greatly benefit from a quantitative approach to the data, including a summary table of the results of the various conditions tested. Please add this important table.
- This reviewer would expect that the endpoint (90 min) would roughly reflect the occupancy results from time-lapse (45 min) experiments. Based on the data presented in Figures 1, 2, S1-3, this does not appear to be the case. 50% of Cse4-GFP and 70% Ndc10-mCherry colocalized with CEN3 DNA in the endpoint experiment, whereas in Fig 2C, ~30 and ~52 traces were positive for Cse4-GFP and Ndc10-mCherry, resp. with the former having drastically lower residency survival compared to Ndc10-mCherry. If indeed, 50% of Cse4-GFP makes it to the endpoint, about 50% of all traces should reach the end of the 45 minutes time-lapse window. Only about 1/3 of all positive Cse4-GFP traces can be seen at the end of the 45 min window. Could this be due to technical issues of photostability of GFP? Why does the colocalization of Cse4 signal with the DNA signal disappear so readily? Are Cse4 so unstable? Is the same true for canonical H3 nucleosomes? This unlikely true for nucleosomes in cells. Along the same lines, in Suppl Fig 3 there is a disconnect between residency survival

and endpoint colocalization on either CEN3, CEN7, or CEN9. What could be the underlying mechanism between the discordance of endpoint results and time-lapse results? Could this be the result of experimental differences?
- What fraction of particles show colocalization between Cse4-GFP and Ndc10-mCherry? What fraction of occupancy time show colocalization between Cse4-GFP and Ndc10-mCherry?
Altogether, understanding the limitation and benefits of endpoint analysis and time-lapse analysis in this particular experimental set-up is important to be able to interpret the results. Please clarify these points.
- Page 9, third sentence of third paragraph it is stated that the "results suggests that Scm3 helps promote more stable binding of Cse4 ...". This is indeed one possible explanation of the results, and this possibility is tested by overexpressing Psh1 or Scm3 by endpoint colocalization analysis. 1) Taking the concerns regarding the endpoint vs time-lapse results into account, wouldn't it be more accurate to compare either time-lapse results against each other or endpoint results? 2) Alternatively, more stable Cse4 particles are able to recruit Scm3, simply because of the increased binding opportunity of a more stable particle. In this scenario, just the residency time of Cse4 alone is the predicting factor in likelihood to associate with Scm3. To test the latter possibility, Cse4 stability would need to be altered. Please consider this experiment- should be relatively easy with the right mutant of either CSE4 or CDEII (see Luger or Wu papers).
- In Figure 1C and Supplemental Figure 5B, there appears to be foci that CEN3-ATTO-647 positive, but Cse4-GFP negative and visa verse. It seems logical that there are DNA molecules that didn't reconstitute Cse4 nucleosomes. But how can there be Cse4-GFP positive foci without a positive DNA signal? Is it possible that unlabeled DNA make it onto the flow chamber? If so, can these unlabeled DNA be visualized by Sytox Orange for instance to confirm that no spurious Cse4 deposition occurred? Please clarify.
- On page 10, at the end of the first paragraph, growth phenotype of pGAL-SCM3 and pGAL-PSH1 mutants were tested. On GAL plates, pGAL-PSH1 shows reduced growth, but not pGAL-SCM3. Wouldn't a more accurate conclusion be that Psh1 is limiting stable centromeric nucleosome formation, instead of Scm3?
- In the section where DNA was tethered at either one or both ends, an important control is missing. How does such a set-up impact nucleosome formation in general. Can H3 nucleosomes form on random DNA that is either tethered at one or both ends? Does this potentially affect the unwrapping potential/topology of AT-tract DNA? Please comment.

Minor comments

- Censored data points are not explained in the text.
- Number of particles tested should be reported in the main and supplemental figures, not just the legends for those readers who first skim the manuscript before deciding to read it.
- Typo on page 5, first line: "nucleosom" should be "nucleosome".
- Typo on page 9, second line: sentence is missing something "... is required for Scm3-dependent ..."
- It is unclear how the difference in Supplemental figure 5D was calculated.
- Figure 4C: why are there more Ndc10-mCherry foci observed in double tethered constructs vs single tethered constructs?
- For the display of individual traces as shown in Fig 2B, 3A, 4E, and 5E, it might be more informative if the z-normalized signal and the binary read-out are shown in separate windows to better appreciate how the z-normalized signal was interpretated.
- Page 17, fifth line of the second paragraph, it is stated that a conserved feature of centromeres is their AT-richness. This is most certainly true for the majority of species studied thus far, but bovine centromeres for instance are about 54% GC rich. Indeed, Melters et al 2013 Genome Biol showed that in certain clades centromeres can be comprised of GC-rich sequences. It might be worthwhile to nuance this statement.
- Page 17, last paragraph. Work by Karolin Luger and Carl Wu is cited in relationship to AT-rich DNA being unfavorable for canonical nucleosome deposition. A citation is missing here: Stormberg & Lyubchenko 2022 IJMS 23(19): 11385. Also, the first person to show that AT-tracts affect nucleosome positioning are Andrew Travers and Drew. This landmark work should be cited.
- Page 18, 9th line from the top, it is stated that yeast centromeres are sensitive to negative genetic drift. This reviewer is of the understanding that genetic drift is a statistical fluctuation of allele frequency, which can result in either gain or loss of specific alleles. Population size is a major factor in the potential power of genetic drift. The smaller a population, the greater the effect. Budding yeast is found large numbers, which would mean that drift only has limited predicted impact. Maybe the authors meant to use the term purifying selection?

2. Significance:

Significance (Required)

This study developed an in vitro imaging technique that allows native proteins from whole cell lysates to associate with a specific DNA sequence that is fixed to a surface. By labeling proteins with specific fluorophore-tags colocalization provides insightful proximity data. By creating mutants, the assembly or disassembly of protein complexes on native or mutated DNAs can therefore be tracked in real time. In a way,

this is a huge leap forward from gel shift EMSA assays that have traditionally been used to do comparative kinetics in biochemistry.

This makes this technique ideal for studying DNA binding complexes, and potentially, even RNA-binding complexes. This study shows both the importance of using genetic mutants, as well as testing the effects of the fixed DNA sequence. As many individual fixed DNA molecules can be tracked at one, it allows for high-throughput analysis, similar to powerful DNA curtain work from Eric Greene's lab. Overall, this is a promising new single-molecule technique that combines in vitro and ex vivo sample sources, and will likely appeal to a broad range of molecular and biophysics scientists.

3. How much time do you estimate the authors will need to complete the suggested revisions:

Estimated time to Complete Revisions (Required)

(Decision Recommendation)

Between 1 and 3 months

4. *Review Commons* values the work of reviewers and encourages them to get credit for their work. Select 'Yes' below to register your reviewing activity at Web of Science Reviewer Recognition Service (formerly Publons); note that the content of your review will not be visible on Web of Science.

Web of Science Reviewer Recognition

Yes

Full Revision

Manuscript number: RC-2023-01847
Corresponding author(s): Biggins, Sue

[Please use this template only if the submitted manuscript should be considered by the affiliate journal as a full revision in response to the points raised by the reviewers.

If you wish to submit a preliminary revision with a revision plan, please use our "Revision Plan" template. ***It is important to use the appropriate template to clearly inform the editors of your intentions.****]*

1. General Statements [optional]

We are grateful for the thoughtful comments and suggestions from the reviewers which we feel have resulted in a manuscript that is both clearer to the reader and more rigorous. We have addressed the suggested revisions point by point below:

2. Point-by-point description of the revisions

Reviewer #1
Major comments:

Figure 2E. This shows the residence time of Cse4 without Ndc10 association. How does this compare to the residence time on mutant CEN3 (Supplemental Figure 1). It looks like Cse4 still binds to CEN3 with some specificity even in the absence of Ndc10. Does this suggest that Cse4 has some intrinsic ability to recognize CEN3? Alternatively, Ndc10 is still required for Cse4 binding but is below detection in the Cse4-alone residence lifetimes. Ideally, the authors would compare this with Cse4 binding in an Ndc10 mutant.

We thank the reviewer for this interesting question. As suggested, we analyzed Cse4 behavior on the mutant $CDEIII^{mut}$ CEN3 DNA, which does not stably recruit Ndc10 (Figure 1C), using the real-time colocalization assay. Although overall Cse4 associations were reduced, we still observed transient interactions, consistent with the possibility that Cse4 has some intrinsic ability to recognize CEN3. Kaplan-Meier analysis of the lifetimes of Cse4 colocalizations on $CDEIII^{mut}$ CEN3 DNA were significantly reduced when compared to CEN DNA (Figure EV1G, H). We have added these points to the text (p. 10, lines 29-31 and p. 11, lines 1-8).

Figure 3 explores the very interesting relationship between Scm3 dynamics and Cse4 binding but I feel that the data is not fully flushed out.
A key finding is that Cse4 can potentially bind to CEN DNA prior to engaging with Scm3 to be incorporated. This predicts that Cse4 should bind first and then colocalize with Scm3. Can this be detected in the timing of the traces? How often does Scm3 bind to already prebound Cse4

and does this correlate with Cse4 residing longer?

We performed new and more rigorous analyses of the data to address these questions in the revised manuscript. After our analysis to calculate ternary^{Scm3} off-rates, we analyzed the relative timing of these ternary residences and found that indeed Cse4 can bind to CEN DNA prior to Scm3 and these events do correlate with Cse4 residing longer. Complete analysis of the binding order of Cse4 and Scm3 ternary residences revealed that Scm3 bound to CEN3 DNA prior to Cse4 more often than Cse4 preceding Scm3 (46% vs 34% of ternary residences) with the remaining 20% arriving simultaneously (Figure EV2E). Despite a difference in prevalence, the median lifetimes of both Scm3-First and Scm3-Last contexts were similar to each other (Figure EV2F) and both were significantly stabilized when compared to non-ternary residences. These results highlight a potential mechanism where Scm3 catalyzes stable Cse4 incorporation at centromeric DNA instead of delivering it to the centromere regardless of the order of arrival. These data are now reported and discussed in the revision (p. 12, line 11-p.13, line 10).

A related and perhaps even more interesting point is whether Scm3 is involved in "loading" of Cse4. If so, then one would expect that once Cse4 is assembled into nucleosomes it should be stable, even if Scm3 leaves. Can the authors extract this from the data? Alternatively, it is possible that Scm3 remains associated to Cse4 to maintain the nucleosome which would imply a more extended role for Scm3 apart from assembly alone. It would be interesting if information on this can be extracted from the data.

Using our updated analysis of ternary^{Scm3} Cse4 residences, feel we have addressed this possibility indirectly in a couple of ways. First, we found that in instances where ternary Scm3/Cse4 complexes are formed, Scm3 co-occupied the CEN DNA an average of 56.0% of the total Cse4 residence time, which is distinctly shorter than the 84% of the total Cse4 residence that was occupied by Ndc10 in Cse4/Ndc10 ternary residences. These findings are consistent with a Scm3-turnover mechanism that occurs post ternary complex formation with Cse4 as we found that Cse4 off-rates were significantly reduced after Scm3 association (Figure 3D).

Second, further analysis of Scm3 residence behavior revealed that there was no significant stabilization of Scm3 after ternary Cse4/Scm3 complex formation vs non-ternary Scm3 residences found in either off-rates (33 hr^{-1} vs 32 hr^{-1}, Figure EV2C) or median lifetimes (45 s vs 52 s, Figure EV2D). These results indicate that Scm3 association is not stabilized like Cse4 after ternary complex formation and points to a potential catalytic role in Cse4 nucleosome formation, leaving a stable Cse4 nucleosome after turnover. We reported these findings in the revision results section (p. 12, lines 11-16) as well as briefly within the discussion.

Even in the presence of Scm3 and CCAN components, Cse4 appears to have a limited lifetime in the in vitro assay compared to in vivo stability. The authors should speculate on whether activities exist in their extract that actively disassembles nucleosomes. Perhaps ATP could be depleted to inactivate remodellers?

Full Revision

This is an excellent suggestion that we addressed with a new experiment. We performed an endpoint localization experiment with lysate containing fluorescently labeled Ndc10 and Cse4 and then removed the lysate and incubated the slide for 24hr in imaging buffer at RT. Strikingly, the proteins were maintained at the CEN DNA with a high protein total colocalization (~75% retention) (shown in Figure EV1B, C). These data suggest that the lysates may contain negative regulatory factors and we have added this point in the revised text (p. 9 lines 6-25).

We were not able to address whether the removal of ATP stabilized the proteins because we previously found that ATP depletion of the lysates completely abrogates kinetochore assembly in extracts. We will need to eventually dissect the role of remodelers in future work using a different approach.

For Figure 6, it is not clear why AT-track mutants of CDEII are labeled as genetically stable and genetically unstable. This is confusing as the "genetically stable" show a more than 10-fold increase in chromosome loss rates. Perhaps these can be renamed into "unstable" and "very unstable" or "weak" and "strong" mutants, just to make clear that these classes are both poorer than wild type.

We had deferred to the nomenclature used in the previous study (Baker and Rogers, 2005) which initially characterized these mutants. To avoid this confusion, we have renamed these mutants “unstable” and “very unstable” as suggested to make it clearer that none of these synthetic mutants fully recapitulate WT CEN3 behavior.

Finally, it would be wonderful to include data to assess whether a full Cse4 nucleosome is assembled or a partial nucleosome e.g. just Cse4/H4 tetrasomes. This could be done by tracking the accumulation of H2A or H2B at the CEN3. This would give further insight into what step Scm3 catalyses.

This is a very interesting suggestion that we were not able to directly address. Epitope tagging of these histone proteins in *Saccharomyces cerevisiae* with endogenous fluorophores has proved challenging due to gene duplication, overall sensitivity to histone levels within the cells and disruption of histone function by epitope tagging. We hope to find a workable method to address this in the future to address this question directly.

Minor comments:

Typo on page 5, line 1 "nucleosom" missing an e.

We have corrected this in the revised text.

Kaplan-Meyer should be spelled Kaplan-Meier

Full Revision

We have corrected this in the revised text.

The term "censored" is mentioned across many figures but comes up just ones in the methods where it is not clearly explained. Perhaps this could be clarified in the legend.

We have now provided a clear explanation of the term “censored” in the text on p. 28, lines 25-27. It has also been added to the figure legends and reported in the *Statistical tests* section of the methods section to address this point.

The abstract states that Cse4 can arrive at the centromere without its chaperone. More likely, Cse4 is in complex with other chaperones that may allow it to bind. Perhaps the abstract can be modified to read "Cse4 can arrive at the centromere without its dedicated centromere-specific chaperone Scm3..."

We updated the abstract to reflect this point in the revised text.

Related to this point, the discussion states the possibility that Cse4 can initially bind to CEN3 via other more general chaperones. However, it should be acknowledged that transient Cse4 binding in their assay may simply occur through mass action due to high concentrations of CEN3 DNA. In vivo, this transient binding may not be that relevant.

We acknowledge this potential caveat in the discussion section (p. 20 lines 15-18), although we feel this is somewhat unlikely due to our observation of significantly reduced Cse4 binding on CDEIII^{mut} DNA despite DNA concentrations being similar to previous assays (Figure EV3A). We speculate that some of this transient behavior is at least in part driven by two major factors: negative regulatory factors within our cellular extracts that counter nucleosome formation (as explored in Figure EV1B-C) and photostability of the endogenous fluorophores used within the study (Figure EV1D-E). These points were highlighted within the second paragraph of page 9.

Reviewer #2
Major comments:

1. Figure S1A-D seem like some of the most compelling data in the paper to bolster the rigor of their experimental setup. There appears to be plenty of space to include these data in the main figure set in Figure 1 after panel D. The authors would be well served to consider moving S1A-D somewhere in the main figure set.

We appreciate the helpful feedback on the importance of the date found in Supplemental Figure 1 and have now incorporated it into Figure 1 within the main text as suggested.

2. The authors conclude that Ndc10 recruits HJURP(Smc3) to the yeast point centromeres. If this is the case, can the TIRFM assay measure ternary residence lifetimes complexes between

Full Revision

Ndc10/HJURP(Smc3)/CenDNA?

We made the conclusion that Ndc10 recruits Scm3 based on previous publications showing this requirement *in vivo*. We have now attempted to address this in our assay indirectly by monitoring Scm3 behavior on the CDEIII^{mut} CEN3 DNA that lacks Ndc10. Surprisingly, we found that Scm3 interacted similarly with CDEIII^{mut} CEN3 DNA and actually showed an increase in median lifetimes vs. CEN3 DNA (Figure EV2B), suggesting its intrinsic DNA-binding activity may be the primary driver of its CEN DNA binding and that stable Cse4 association is required for its turnover. These data suggest that Ndc10 is not driving Scm3 interaction (or targeting) to CEN3. We are grateful to the reviewer for pointing this out and have adjusted our conclusions in the revised manuscript (p. 12, line 26-p.13 line 10).

3. Throughout the manuscript short- and long- term residence lifespans are mentioned, referencing the figures containing lines with lengths depicting residence times. This is a qualitative reference to short and long residences. Can the authors provide a graph for short-term (<120 s) and long-term (> 300 s) residence life-spans for, CENPA alone, CENPA/Ndc10, and CENPA/HJURP on CEN3 DNA? Or some figure similar to Figure 3C, but reporting the proportion of short-term vs long-term residence?

We typically used Kaplan-Meier survival function estimates to compare binding behavior but agree that quantification of residences within these contexts may be easier for the reader to follow. We have therefore quantified short-term (<120 s) and long-term (> 300 s) as suggested and added them as a panel (F) to Figure EV1 and as panel (E) in Figure 3.

4. The choice of CCAN components for analysis in Fig. 5 is interesting, but many readers may be curious why Mif2 wasn't selected for disruption, since it has such a cozy placement with CENP-A and CEN DNA. Can choice to not include Mif2 mutants/degrons be mentioned/justified in the text (unless, even better yet, they choose to address Mif2 role directly in new experimentation)?

We relied on structural models to choose CCAN proteins that are in close proximity to the DNA. Because Mif2 is not in these structures, we did not include it in our studies. We have explained this in the revised text (p. 16 lines 14-17) and agree it is an interesting future area of study.

Minor comments:

5. Are these whole cell extracts (WCE) DNA-free? I'm curious if there is any competition from endogenous DNA from the yeast cellular extract.

The extracts are not DNA-free so it is likely there is some competition from endogenous DNA. We have avoided enzymatically removing the DNA since the TIRF assay depends on the integrity of DNA.

6. In relation to Mif2 and comment #4 above, do the authors make any connection to their results with synthetic nucleosome sequences not being conducive to yeast centromere formation with the prior observation (Allu et al 2019) using recombinant components that the human version of Mif2 more easily saturates its binding sites on CENP-A nucleosomes when they are assembled with natural centromere DNA rather than the Widom 601 sequence?

We did not speculate on the role of Mif2 and stability of synthetic nucleosome sequences. This is an interesting point but the differences between the yeast and human systems combined with the fact we have not yet started to study Mif2 made it seem too premature to include in this manuscript.

7. Providing a gel (or other measure) of the DNA templates (750, 250, and 80 bp) used in TIRFM assay would be nice to show to confirm the designed size of the pre-tethered DNA.

We agree this is a helpful control and we have now included it as a panel in Figure EV5B.

8. Some of the references to figures/figure panels in the main text do not match the figures. (discussion, pg 16, paragraph 1 & 2;pg 18, paragraph 1).

We have updated references mentioned to reflect to the correct figure in both sections of the discussion.

Reviewer #3
Major comments:
-->Statistics are highly recommended for all the data in the ms.

We have included log-rank analysis to instances where two survival functions were plotted together where appropriate. P-values for all these analyses were reported in the appropriate figure legends.

+ At what rate is data collected in the TIRFM setup. For clarity for the reader, it is important to provide imaging details for time-lapse. What is the impact of photobleaching on the stability of the fluorophore signal? Please clarify.

This is a helpful suggestion and we have now included imaging details (like time intervals for each channel) when the real-time assay is introduced in the results section (p. 8, lines 10-12). We have also provided additional details for the photobleaching estimates and how these might censor data in turn (p. 9, lines 14-25). Although photobleaching is a primary limitation of the time-lapse assays, we point out that it is appropriate to compare protein behavior under identical imaging parameters within differing contexts. We also noted that we typically compared time-

lapse behavior (which is affected by photobleaching) with endpoint assays to ensure consistent behavior.

+ The power of single-molecule technique is precisely that such data can be made quantitative. Indeed, the Kaplan-Meyer graphs do show nice quantitative results. Unfortunately, in the text few quantitative measurements are reported. In fact, the Kaplan-Meyer graphs can be interpreted in a quantitative manner such as probability of residency time. Although in most cases the statistical significance between two conditions can be expected, this is not formally calculated and shown. What is the 50% survival time of Cse4 alone or with Ndc10, for instance? This manuscript would greatly benefit from a quantitative approach to the data, including a summary table of the results of the various conditions tested. Please add this important table.

We initially put the quantitative data in the figure legends but omitted it from the main text for simplicity but appreciate the Reviewer's point. We note that we performed log-rank tests on all Kaplan-Meier analyses that are plotted on the same graph to provide statistical differences where applicable and have included all P-values in the figure legends. In response to the suggestion, we have now also included a table (Table 1) that contains the median survival time for all proteins tested as well as the median survival times for the differing contexts tested for quick reference and easier comparison for the reader.

+ This reviewer would expect that the endpoint (90 min) would roughly reflect the occupancy results from time-lapse (45 min) experiments. Based on the data presented in Figures 1, 2, S1-3, this does not appear to be the case. 50% of Cse4-GFP and 70% Ndc10-mCherry colocalized with CEN3 DNA in the endpoint experiment, whereas in Fig 2C, ~30 and ~52 traces were positive for Cse4-GFP and Ndc10-mCherry, resp. with the former having drastically lower residency survival compared to Ndc10-mCherry. If indeed, 50% of Cse4-GFP makes it to the endpoint, about 50% of all traces should reach the end of the 45 minutes time-lapse window. Only about 1/3 of all positive Cse4-GFP traces can be seen at the end of the 45 min window. Could this be due to technical issues of photostability of GFP? Why does the colocalization of Cse4 signal with the DNA signal disappear so readily? Are Cse4 so unstable? Is the same true for canonical H3 nucleosomes? This unlikely true for nucleosomes in cells.

This is a valid concern, and we appreciate the thoughtful critique. The inconsistency noted between the initial endpoint colocalization and those reported later in the paper is mainly due to the difference between yeast strains carrying Cse4 tagged alone in comparison to multiple kinetochore proteins with tags that exhibit mild genetic interactions. This point is now explained in the revised text (p. 8, line 29-p. 9. line 3).

Photostability is also a factor in the live imaging experiments compared to the endpoint localization assays. However, our photobleaching estimates suggest that the Cse4 lifetimes are not limited by photobleaching (Figure EV1D, E) so we do not believe that accounts for the differences between experiments and it is mainly the presence of multiple epitope tags.

Full Revision

In regard to why Cse4 is not more stable, Reviewer 1 had the same question so we performed an experiment to address whether the lysate contains negative regulatory factors. We found that Cse4 is stable once the lysate is removed (Figure EV1B, C), consistent with the idea that there are factors that disrupt it in the lysate. We discuss these potential reasons for transient association in the revised text (p. 9, lines 4-25).

It should also be noted that there are clear differences in nucleosome formation in reconstitutions and within our extracts, as evident by the Widom-601 DNA data (Figure 6D). This was not necessarily unexpected, as extracts are a much more complex medium, but we are encouraged by the fact that at least once formed, these Cse4-containing particles on CEN DNA are perhaps more stable than their reconstituted counterparts that seem to be so far unsuitable for structural studies.

Along the same lines, in Suppl Fig 3 there is a disconnect between residency survival and endpoint colocalization on either CEN3, CEN7, or CEN9. What could be the underlying mechanism between the discordance of endpoint results and time-lapse results? Could this be the result of experimental differences?

We are grateful that this discrepancy was highlighted to us, as upon closer examination we discovered that endpoint colocalization analysis had not been correctly updated in the figure to include data from equivalent genetic backgrounds as the CEN3 and CEN9 assays. Updating the figure in Appendix Figure S2 to include this data remedied this discrepancy.

+ What fraction of particles show colocalization between Cse4-GFP and Ndc10-mCherry? What fraction of occupancy time show colocalization between Cse4-GFP and Ndc10-mCherry? Altogether, understanding the limitation and benefits of endpoint analysis and time-lapse analysis in this particular experimental set-up is important to be able to interpret the results. Please clarify these points.

We have now added particle numbers to all survival estimate plots which makes it much easier for the reader to interpret the proportion of Cse4 residences that are ternary vs. non-ternary in instances where off-rates were quantified and Kaplan-Meier analysis was performed on the resulting lifetimes. We determined that for ternary Cse4-Ndc10 residences, Cse4 and Ndc10 co-occupied the CEN DNA an average of ~84% of the total Cse4 residence.

+ Page 9, third sentence of third paragraph it is stated that the "results suggests that Scm3 helps promote more stable binding of Cse4 ...". This is indeed one possible explanation of the results, and this possibility is tested by overexpressing Psh1 or Scm3 by endpoint colocalization analysis. 1) Taking the concerns regarding the endpoint vs time-lapse results into account, wouldn't it be more accurate to compare either time-lapse results against each other or endpoint results? 2) Alternatively, more stable Cse4 particles are able to recruit Scm3, simply because of the increased binding opportunity of a more stable particle. In this scenario, just the residency time of Cse4 alone is the predicting factor in likelihood to associate with Scm3. To test the latter

possibility, Cse4 stability would need to be altered. Please consider this experiment- should be relatively easy with the right mutant of either CSE4 or CDEII (see Luger or Wu papers).

We appreciate the points raised here and addressed both as follows. For point (1) we altered the text in the third paragraph of the section, *The conserved chaperone Scm3^{HJURP} is a limiting cofactor that promotes stable association Cse4$^{CENP\text{-}A}$ with the centromere,* to make it more clear to the reader that in the experiments presented in Figure EV4, endpoint analysis results were only compared to each other, and likewise time-lapse experiments were only compared to each other for each genetic background. While the results were consistent between experiments, we did not directly compare results from one to the results of another, but instead we used both assays to characterize Cse4$^{CENP\text{-}A}$ behavior more completely in differing contexts.

To test the alternative hypothesis proposed in point (2), we sought to avoid potential selection bias by utilizing off-rate analysis, which enabled us to separate the portions of Cse4 residences that occurred prior to ternary association with either Ndc10 or Scm3. This unbiased approach allowed us to compare Cse4 residence lifetimes pre and post ternary association and we found that there were still significant differences in off-rates and median lifetimes of the associated ternary and non-ternary residences using this updated analysis. We thank the reviewer for helping to guide us towards this more robust analysis.

Based on the recommendation in point (2), we also sought to directly compare the behavior of Cse4 and Scm3 on the "Very Unstable" CDEII mutants described in the section, *DNA-composition of centromeres contributes to genetic stability through Cse4$^{CENP\text{-}A}$ recruitment*. In this case, equivalent extracts were used and Cse4 stability was altered directly via the DNA template. When the off rates of ternary^{Scm3} Cse4 residences were compared, we found a significant increase in off-rates of Cse4 on the "Very Unstable" CDEII mutant CEN DNA (Appendix Figure S3B) compared to WT CEN DNA. If the alternative hypothesis proposed in point (2) were true, we would expect this reduction in median lifetime to correlate with a lower proportion of Cse4-Scm3 ternary association but quantification yielded proportions that, while varied, were not on average lower than the proportion of Cse4-Scm4 association on CEN3 DNA (.23 vs .31, Appendix Figure S3A). This finding, taken together with the fact that it would be difficult for us to propose an alternative hypothesis that explains the results outlined in Figure EV4, supports our hypothesis that Scm3 helps promote more stable binding of Cse4 and that this stabilization is directly influenced by DNA sequence composition.

+ In Figure 1C and Supplemental Figure 5B, there appears to be foci that CEN3-ATTO-647 positive, but Cse4-GFP negative and visa verse. It seems logical that there are DNA molecules that didn't reconstitute Cse4 nucleosomes. But how can there be Cse4-GFP positive foci without a positive DNA signal? Is it possible that unlabeled DNA make it onto the flow chamber? If so, can these unlabeled DNA be visualized by Sytox Orange for instance to confirm that no spurious Cse4 deposition occurred? Please clarify.

Full Revision

Because it is unlikely that random associations will colocalize with the labeled DNA based on control assays (Supp. Figure 1C) that show this occurs rarely (<3%), it is more likely that some small percentage of DNA lack an active fluorophore within our flow chambers due to photobleaching prior to imaging. While this cannot be completely avoided, we exclude any colocalization that occurs outside of labeled DNA foci, so these spots do not affect the dataset. It is also possible that during incubation in WCE, occasional non-specific association of proteins or debris occurs on the coverslips despite their passivation. It is unclear if such particles are Cse4-GFP or some other object that is auto-fluorescent at 488nm wavelength.

+ On page 10, at the end of the first paragraph, growth phenotype of pGAL-SCM3 and pGAL-PSH1 mutants were tested. On GAL plates, pGAL-PSH1 shows reduced growth, but not pGAL-SCM3. Wouldn't a more accurate conclusion be that Psh1 is limiting stable centromeric nucleosome formation, instead of Scm3?

The growth defects on galactose don't necessarily mean that a factor is limiting in cells. Instead, they report on whether changing the relative amounts of the complex lead to phenotypes in cells that could be the result of many causes that would require characterization of the phenotypes to understand. In this case, we presume that Psh1 titrates Scm3 away from Cse4 to prevent nucleosome formation in vivo. However, we have not directly tested this so we just concluded that the relative levels of the complexes are important for cell growth.

+ In the section where DNA was tethered at either one or both ends, an important control is missing. How does such a set-up impact nucleosome formation in general. Can H3 nucleosomes form on random DNA that is either tethered at one or both ends? Does this potentially affect the unwrapping potential/topology of AT-tract DNA? Please comment.

This is an interesting point and one that we hope to explore further in the future but was beyond the scope of this paper. We suspect that restrictions via tethering would also limit canonical nucleosome formation on random DNA. We envision that unwrapping may be affected as well and hope to explore this via other, potentially better suited techniques like optical tweezers.

Minor comments

+ Censored data points are not explained in the text.

A brief explanation of censorship was added to the figure legends and we have now provided a clear explanation of the term "censored" in the text on p. 28, lines 25-27. It has also been reported in the *Statistical tests* section of the methods section to address this point.

+ Number of particles tested should be reported in the main and supplemental figures, not just the legends for those readers who first skim the manuscript before deciding to read it.

We add these values to all Kaplan-Meier plots in all figures (Main, Expanded View, and Appendix)

Full Revision

+ Typo on page 5, first line: "nucleosom" should be "nucleosome".

We fixed this in the text.

+ Typo on page 9, second line: sentence is missing something "... is required for Scm3-dependent ..."

We fixed this in the text.

+ It is unclear how the difference in Supplemental figure 5D was calculated.

We included log-rank test generated P-values as well as description in the figure legend of EV4.

+ Figure 4C: why are there more Ndc10-mCherry foci observed in double tethered constructs vs single tethered constructs?

There can be variances in DNA density between slides, particularly with non-dye labeled DNA template. We updated figure panel C to include a representative image with similar Ndc10 density.

+ For the display of individual traces as shown in Fig 2B, 3A, 4E, and 5E, it might be more informative if the z-normalized signal and the binary read-out are shown in separate windows to better appreciate how the z-normalized signal was interpretated.

Due to spacing limits within figures we attempted to accommodate this by reducing the thickness of the binary read-out and ensured that the raw data traces were overlaid for easier interpretation by the reader.

+ Page 17, fifth line of the second paragraph, it is stated that a conserved feature of centromeres is their AT-richness. This is most certainly true for the majority of species studied thus far, but bovine centromeres for instance are about 54% GC rich. Indeed, Melters et al 2013 Genome Biol showed that in certain clades centromeres can be comprised of GC-rich sequences. It might be worthwhile to nuance this statement.

We have updated the text to reflect that AT-rich DNA is widely conserved but not a universal feature of centromeres.

+ Page 17, last paragraph. Work by Karolin Luger and Carl Wu is cited in relationship to AT-rich DNA being unfavorable for canonical nucleosome deposition. A citation is missing here: Stormberg & Lyubchenko 2022 IJMS 23(19): 11385. Also, the first person to show that AT-tracts affect nucleosome positioning are Andrew Travers and Drew. This landmark work should be cited.

Full Revision

We thank the Reviewer for noticing this and have added the appropriate citations.

+ Page 18, 9th line from the top, it is stated that yeast centromeres are sensitive to negative genetic drift. This reviewer is of the understanding that genetic drift is a statistical fluctuation of allele frequency, which can result in either gain or loss of specific alleles. Population size is a major factor in the potential power of genetic drift. The smaller a population, the greater the effect. Budding yeast is found large numbers, which would mean that drift only has limited predicted impact. Maybe the authors meant to use the term purifying selection?

We appreciate this clarification and agree with the reviewer, we have updated the manuscript to cite purifying selection and not genetic drift as at centromeres.

Dr. Sue Biggins
Fred Hutchinson Cancer Center
Division of Basic Sciences
1100 Fairview Ave N
Mailstop A2-168, PO BOx 19024
Seattle, WA 98109

23rd Jun 2023

Re: EMBOJ-2023-114534
Direct observation of coordinated assembly of individual native centromeric nucleosomes

Dear Sue,

Thank you again for submitting your revised Review Commons manuscript for consideration by The EMBO Journal. In light of the supportive original comments and the already included modifications in response to them, I decided to treat this study like a regular revision, and sent it directly back to two of the original referees. As you will see from their feedback copied below, both of them are fully satisfied with your responses and consider the manuscript now ready for publication. Before proceeding with formal acceptance, I would therefore only need to ask for a few remaining editorial modifications, as follows:

- Pre-acceptance checks by our data editors have raised a few queries with the data descriptors in the figure legends, which you will find as comments in the attached edited/commented Word document with activated "Track changes" option. I would appreciate if you incorporated the requested final text modifications and answered the Figure legend queries directly in this version (and modified figures where necessary), uploading the edited main text document upon resubmission with changes/additions still highlighted via the "Track changes" option, to facilitate our final checking.

- As we are switching from a free-text author contribution statement towards a more formal statement based on Contributor Role Taxonomy (CRediT) terms, please specify each author's contribution(s) directly in the Author Information page of our submission system during upload of the final manuscript, and remove the current Author Contribution section in the text. See https://casrai.org/credit/ for more information.

- In the Appendix, please make sure to rename the included tables into "Appendix Table S1/2/3" in all instances.

- Please convert all movies into "Expanded View movies", renaming them "Movie EV1/2/3..." and making sure to reference each one of them at least once in the text. Also, please cut the movie legends from the Appendix file, instead placing each one into one separate legend text file per EV movie; then move each legend txt file together with the respective movie file into one separate ZIP archive per movie, before re-uploading these ZIP files as "Movie EV1/2/3..."

- Finally, please provide suggestions for a short 'blurb' text prefacing and summing up the conceptual aspect of the study in two sentences (max. 250 characters), followed by 3-5 one-sentence 'bullet points' with brief factual statements of key results of the paper; they will form the basis of an editor-written 'Synopsis' accompanying the online version of the article. Please also upload a synopsis image, which can be used as a "visual title" for the synopsis section of your paper (maybe a much-simplified version of Figure 7?). The image should be in PNG or JPG format, and please make sure that it remains in the modest dimensions of (exactly) 550 pixels wide and 300-600 pixels high.

I am therefore returning the manuscript to you for a final round of minor revision, to allow you to make these adjustments and upload all modified files. Once we will have received them, we should be ready to swiftly proceed with formal acceptance and production of the manuscript.

With kind regards,

Hartmut

Hartmut Vodermaier, PhD
Senior Editor, The EMBO Journal
h.vodermaier@embojournal.org

Further information is available in our Guide For Authors:
https://www.embopress.org/page/journal/14602075/authorguide

In the interest of ensuring the conceptual advance provided by the work, we recommend submitting a revision within 3 months (21st Sep 2023). Please discuss the revision progress ahead of this time with the editor if you require more time to complete the revisions. Use the link below to submit your revision:

Link Not Available

Referee #1:

The authors have made an extensive effort to address my earlier questions and concerns. The addition of a more thorough analysis of Cse4 and Scm3 residence times to determine to what extent Cse4 arrives prior to Scm3 and how Scm3 impacts Cse4 stability is particularly insightful. Also, the new experiment to determine the intrinsic stability of Ndc10 and Cse4 complexes (in the absence of extract) is a great addition to the paper. This is an excellent paper that is novel, well-executed, and offers new insight. This manuscript is ideally suited for the EMBO Journal.

Referee #3:

The manuscript "Single molecule visualization of native centromeric nucleosome formation reveals coordinated deposition by kinetochore proteins and centromere DNA sequence" by Popchock and colleagues describes a new high-throughput single-molecule technique that combines both in vitro and in vivo sample sources. Budding yeast centromeres are genetically defined centromeres, which makes them ideal for studying short DNA segments at the single-molecule level. By flowing in whole cell lysates, Cse4 nucleosomes can form under near physiological conditions. Two analytical experiments were performed: endpoint and time-lapse. In the former case, nucleosomes were allowed to form within 90 minutes and the latter case, nucleosome formation was tracked for up to 45 minutes. In addition, well described genetic mutants were used to assess the stability of Cse4 nucleosomes, as well as different DNA sequences. Overall, this is a very interesting technique with potential to be useful for studying any DNA-based effect, ranging from DNA repair to kinetochore assembly. This manuscript has been reviewed via Review Commons earlier and the authors have addressed all concerns adequately: a table with quantitative results was added; experimental results were added to test whether more stable Cse4 particles more efficiently recruited Scm3; and textual changes were incorporated which improve clarity and completeness of the experimental procedures and discuss technical limitations. We have no further suggestions, and recommend accepting this manuscript.

We congratulate the authors on a very nice set of experiments which significantly contribute to the evolving conversation on dynamics of centromeric nucleosomes.

All editorial and formatting issues were resolved by the authors.

Dr. Sue Biggins
Fred Hutchinson Cancer Center
Division of Basic Sciences
1100 Fairview Ave N
Mailstop A2-168, PO BOx 19024
Seattle, WA 98109

4th Jul 2023

Re: EMBOJ-2023-114534R
Direct observation of coordinated assembly of individual native centromeric nucleosomes

Dear Sue,

Thank you for submitting your final revised manuscript for our consideration. I am pleased to inform you that we have now accepted it for publication in The EMBO Journal.

Before proceeding with production, there is only one final thing - could you please email us another version of the SYNOPSIS IMAGE?
As mentioned in my last email, we need this in 550 pixel width and at least 300 pixel in height (up to 600) - I tried downscaling the much larger image you had uploaded, but I am afraid it always turned out very pixelated and coarse. Hopefully, it should be easier for you, still having the original image files used to prepare the schematics.

Your article will be processed for publication in The EMBO Journal by EMBO Press and Wiley, who will contact you with further information regarding production/publication procedures and license requirements. You will also be provided with page proofs after copy-editing and typesetting of main manuscript and expanded view figure files.

Should you be planning a Press Release on your article, please get in contact with embojournal@wiley.com as early as possible, in order to coordinate publication and release dates.

Thank you again for this contribution to The EMBO Journal and congratulations on a successful publication! Please consider us again in the future for your most exciting work.

With best regards,

Hartmut

Hartmut Vodermaier, PhD
Senior Editor, The EMBO Journal
h.vodermaier@embojournal.org

EMBO Press Author Checklist

Corresponding Author Name: Sue Biggins	
Journal Submitted to: The EMBO Journal	
Manuscript Number: EMBOJ-2023-114534	

USEFUL LINKS FOR COMPLETING THIS FORM
The EMBO Journal - Author Guidelines
EMBO Reports - Author Guidelines
Molecular Systems Biology - Author Guidelines
EMBO Molecular Medicine - Author Guidelines

Reporting Checklist for Life Science Articles (updated January
This checklist is adapted from Materials Design Analysis Reporting (MDAR) Checklist for Authors. MDAR establishes a minimum set of requirements in transparent reporting in the life sciences (see Statement of Task: 10.31222/osf.io/9sm4x). Please follow the journal's guidelines in preparing your
Please note that a copy of this checklist will be published alongside your article.

Abridged guidelines for figures
1. Data
The data shown in figures should satisfy the following conditions:
- the data were obtained and processed according to the field's best practice and are presented to reflect the results of the experiments in an accurate and unbiased manner.
- ideally, figure panels should include only measurements that are directly comparable to each other and obtained with the same assay.
- plots include clearly labeled error bars for independent experiments and sample sizes. Unless justified, error bars should not be shown for technical
- if n<5, the individual data points from each experiment should be plotted. Any statistical test employed should be justified.
- Source Data should be included to report the data underlying figures according to the guidelines set out in the authorship guidelines on Data

2. Captions
Each figure caption should contain the following information, for each panel where they are relevant:
- a specification of the experimental system investigated (eg cell line, species name).
- the assay(s) and method(s) used to carry out the reported observations and measurements.
- an explicit mention of the biological and chemical entity(ies) that are being measured.
- an explicit mention of the biological and chemical entity(ies) that are altered/varied/perturbed in a controlled manner.
- the exact sample size (n) for each experimental group/condition, given as a number, not a range;
- a description of the sample collection allowing the reader to understand whether the samples represent technical or biological replicates (including how many animals, litters, cultures, etc.).
- a statement of how many times the experiment shown was independently replicated in the laboratory.
- definitions of statistical methods and measures:
  - common tests, such as t-test (please specify whether paired vs. unpaired), simple χ^2 tests, Wilcoxon and Mann-Whitney tests, can be unambiguously identified by name only, but more complex techniques should be described in the methods section;
  - are tests one-sided or two-sided?
  - are there adjustments for multiple comparisons?
  - exact statistical test results, e.g., P values = x but not P values < x;
  - definition of 'center values' as median or average;
  - definition of error bars as s.d. or s.e.m.

Please complete ALL of the questions below.
Select "Not Applicable" only when the requested information is not relevant for your study.

Materials

Newly Created Materials	Information included in the manuscript?	In which section is the information available? (Reagents and Tools Table, Materials and Methods, Figures, Data Availability Section)
New materials and reagents need to be available; do any restrictions apply?	Not Applicable	

Antibodies	Information included in the manuscript?	In which section is the information available? (Reagents and Tools Table, Materials and Methods, Figures, Data Availability Section)
For **antibodies** provide the following information:<br>- Commercial antibodies: RRID (if possible) or supplier name, catalogue number and or/clone number<br>- Non-commercial: RRID or citation	Yes	Materials and Methods

DNA and RNA sequences	Information included in the manuscript?	In which section is the information available? (Reagents and Tools Table, Materials and Methods, Figures, Data Availability Section)
**Short novel DNA or RNA including primers, probes**: provide the sequences.	Yes	Appendix Table 3

Cell materials	Information included in the manuscript?	In which section is the information available? (Reagents and Tools Table, Materials and Methods, Figures, Data Availability Section)
**Cell lines:** Provide species information, strain. Provide accession number in repository **OR** supplier name, catalog number, clone number, and/**OR** RRID.	Yes	Appendix Table 2-3
**Primary cultures:** Provide species, strain, sex of origin, genetic modification status.	Not Applicable	
Report if the cell lines were recently **authenticated** (e.g., by STR profiling) and tested for mycoplasma contamination.	Not Applicable	

Experimental animals	Information included in the manuscript?	In which section is the information available? (Reagents and Tools Table, Materials and Methods, Figures, Data Availability Section)
**Laboratory animals or Model organisms:** Provide species, strain, sex, age, genetic modification status. Provide accession number in repository **OR** supplier name, catalog number, clone number, **OR** RRID.	Not Applicable	
**Animal observed in or captured from the field:** Provide species, sex, and age where possible.	Not Applicable	
Please detail **housing and husbandry conditions**.	Not Applicable	

Plants and microbes	Information included in the manuscript?	In which section is the information available? (Reagents and Tools Table, Materials and Methods, Figures, Data Availability Section)
**Plants:** provide species and strain, ecotype and cultivar where relevant, unique accession number if available, and source (including location for collected wild specimens).	Not Applicable	
**Microbes:** provide species and strain, unique accession number if available, and source.	Not Applicable	

Human research participants	Information included in the manuscript?	In which section is the information available? (Reagents and Tools Table, Materials and Methods, Figures, Data Availability Section)
If collected and within the bounds of privacy constraints report on age, sex and gender or ethnicity for all study participants.	Not Applicable	

Core facilities	Information included in the manuscript?	In which section is the information available? (Reagents and Tools Table, Materials and Methods, Figures, Data Availability Section)
If your work benefited from core facilities, was their service mentioned in the acknowledgments section?	Yes	Acknowledgements

Design

Study protocol	Information included in the manuscript?	In which section is the information available? (Reagents and Tools Table, Materials and Methods, Figures, Data Availability Section)
If study protocol has been **pre-registered, provide DOI in the manuscript**. For clinical trials, provide the trial registration number **OR** cite DOI.	Not Applicable	
Report the **clinical trial registration number** (at ClinicalTrials.gov or equivalent), where applicable.	Not Applicable	

Laboratory protocol	Information included in the manuscript?	In which section is the information available? (Reagents and Tools Table, Materials and Methods, Figures, Data Availability Section)
Provide DOI OR other citation details if **external detailed step-by-step protocols** are available.	Not Applicable	

Experimental study design and statistics	Information included in the manuscript?	In which section is the information available? (Reagents and Tools Table, Materials and Methods, Figures, Data Availability Section)
Include a statement about **sample size** estimate even if no statistical methods were used.	Yes	Figures
Were any steps taken to minimize the effects of subjective bias when allocating animals/samples to treatment (e.g. **randomization procedure**)? If yes, have they been described?	Not Applicable	
Include a statement about **blinding** even if no blinding was done.	Not Applicable	
Describe **inclusion/exclusion criteria** if samples or animals were excluded from the analysis. Were the criteria pre-established?<br><br>If sample or data points were omitted from analysis, report if this was due to attrition or intentional exclusion and provide justification.	Not Applicable	
For every figure, are **statistical tests** justified as appropriate? Do the data meet the assumptions of the tests (e.g., normal distribution)? Describe any methods used to assess it. Is there an estimate of variation within each group of data? Is the variance similar between the groups that are being statistically compared?	Yes	Figures, Materials and Methods, Statistical Tests

Sample definition and in-laboratory replication	Information included in the manuscript?	In which section is the information available? (Reagents and Tools Table, Materials and Methods, Figures, Data Availability Section)
In the figure legends: state number of times the experiment was **replicated** in laboratory.	Yes	Figures
In the figure legends: define whether data describe **technical or biological replicates**.	Yes	Figures

Ethics

Ethics	Information included in the manuscript?	In which section is the information available? (Reagents and Tools Table, Materials and Methods, Figures, Data Availability Section)
Studies involving **human participants**: State details of **authority granting ethics approval** (IRB or equivalent committee(s), provide reference number for approval.	Not Applicable	
Studies involving **human participants**: Include a statement confirming that **informed consent** was obtained from all subjects and that the experiments conformed to the principles set out in the WMA Declaration of Helsinki and the Department of Health and Human Services Belmont Report.	Not Applicable	
Studies involving **human participants:** For publication of **patient photos**, include a statement confirming that consent to publish was obtained.	Not Applicable	
Studies involving experimental **animals**: State details of **authority granting ethics approval** (IRB or equivalent committee(s), provide reference number for approval. Include a statement of compliance with ethical regulations.	Not Applicable	
Studies involving **specimen and field samples:** State if relevant **permits** obtained, provide details of authority approving study; if none were required, explain why.	Not Applicable	

Dual Use Research of Concern (DURC)	Information included in the manuscript?	In which section is the information available? (Reagents and Tools Table, Materials and Methods, Figures, Data Availability Section)
Could your study fall under dual use research restrictions? Please check biosecurity documents and list of **select agents and toxins** (CDC): https://www.selectagents.gov/sat/list.htm	Not Applicable	
If you used a select agent, is the security level of the lab appropriate and reported in the manuscript?	Not Applicable	
If a study is subject to dual use research of concern regulations, is the name of the **authority granting approval and reference number** for the regulatory approval provided in the manuscript?	Not Applicable	

Reporting

The MDAR framework recommends adoption of discipline-specific guidelines, established and endorsed through community initiatives. Journals have their own policy about requiring specific guidelines and recommendations to complement MDAR.

Adherence to community standards	Information included in the manuscript?	In which section is the information available? (Reagents and Tools Table, Materials and Methods, Figures, Data Availability Section)
State if relevant guidelines or checklists (e.g., **ICMJE, MIBBI, ARRIVE, PRISMA**) have been followed or provided.	Not Applicable	
For **tumor marker prognostic studies,** we recommend that you follow the **REMARK** reporting guidelines (see link list at top right). See author guidelines, under 'Reporting Guidelines'. Please confirm you have followed these guidelines.	Not Applicable	
For **phase II and III randomized controlled trials**, please refer to the **CONSORT** flow diagram (see link list at top right) and submit the CONSORT checklist (see link list at top right) with your submission. See author guidelines, under 'Reporting Guidelines'. Please confirm you have submitted this list.	Not Applicable	

Data Availability

Data availability	Information included in the manuscript?	In which section is the information available? (Reagents and Tools Table, Materials and Methods, Figures, Data Availability Section)
Have **primary datasets** been deposited according to the journal's guidelines (see 'Data Deposition' section) and the respective accession numbers provided in the Data Availability Section?	Yes	Data Availability Section, Zenodo, Github
Were **human clinical and genomic datasets** deposited in a public access-controlled repository in accordance to ethical obligations to the patients and to the applicable consent agreement?	Not Applicable	
Are **computational models** that are central and integral to a study available without restrictions in a machine-readable form? Were the relevant accession numbers or links provided?	Not Applicable	
If publicly available data were reused, provide the respective **data citations in the reference list**.	Not Applicable	
